# A multidimensional quiet quitting scale: Development and test of a measure of quiet quitting

**DOI:** 10.1371/journal.pone.0317624

**Published:** 2025-04-15

**Authors:** Pankaj C. Patel, Maria João Guedes, Daniel G. Bachrach, Younsung Cho

**Affiliations:** 1 Department of Management and Operations, Villanova School of Business, Villanova University, Villanova, Pennsylvania, United States of America; 2 Department of Management, ISEG - Lisbon School of Economics & Management, University of Lisbon, Lisbon, Portugal; 3 Department of Management, Culverhouse College of Business, The University of Alabama, Tuscaloosa, Alabama, United States of America; University of Trieste: Universita degli Studi di Trieste, ITALY

## Abstract

Growing attention has focused on employees’ repeated workplace decision to engage in work-related tasks and responsibilities at a minimum level that will not lead to their dismissal. This pattern of employee work behavior, labeled “Quiet Quitting,” represents a potentially problematic organizational phenomenon, particularly given increasingly complex work demands that can lower organizational performance. However, the literature lacks both a conceptually anchored definition of the Quiet Quitting construct and an associated empirically validated measure. Through four studies (sample of graduate students, Prolific sample, snowball sample from participants around the world, and a field sample of employees), we develop and validate a two-dimensional Quiet Quitting scale. This scale will facilitate measurement of the construct and development of a more nuanced understanding of its nomological network, correlates, and consequences. Implications for theory and research bearing on employee Quiet Quitting and employee work contributions are offered.

## Introduction

Accelerated by ubiquitous Covid-driven workforce virtualization [1,2], huge numbers of workers in both the United States [3] and around the world [4,5] have become more emotionally detached from their places of work. This increasing emotional detachment stems from multiple intersecting factors. First, pandemic-driven workplace virtualization has disrupted traditional social bonds and organizational identification processes [6], with remote work creating physical and psychological distance from organizational culture and workplace relationships [7]. Second, intensified job demands during global disruption have accelerated employee burnout and emotional exhaustion [8], leading many to psychologically withdraw as a protective mechanism. Third, shifting generational values and expectations about work-life boundaries have challenged traditional assumptions about organizational attachment [9]. Fourth, the rapid transformation of work practices has created role ambiguity and reduced organizational embeddedness [10], weakening emotional connections to work. Finally, increased job insecurity and economic uncertainty have prompted employees to reassess their psychological investment in work [11].

The global physical and emotional disruption introduced by Covid-driven organization-level reorganizations has revealed an increasing prevalence of experiential remoteness among employees, across a broad cross-section of industries [12–16]. Not surprisingly, in light of these shifts away from work [17], a great deal of academic [15,16,18,19] and widespread popular press attention [20–22] has recently focused on the phenomenon. Escalating levels of professional workplace indifference and withdrawal have been primarily anchored among Millennials and Generation Z [23,24]. The phenomenon has earned the popular moniker “Quiet Quitting” [25–27].

The emergence of quiet quitting as a widespread workplace phenomenon reflects fundamental shifts in how employees view their relationship with work and their employers. This behavioral pattern has garnered significant attention not only due to post-pandemic workplace changes but also because it represents a broader questioning of traditional work norms and expectations. Organizations’ increasing reliance on discretionary employee effort, coupled with growing workforce concerns about work-life balance, mental health, and sustainable career development, have created conditions where quiet quitting resonates across industries and organizational levels. The phenomenon’s widespread recognition - from viral social media discussions to executive boardrooms - suggests it captures a fundamental tension in contemporary employment relationships: how much effort beyond minimal requirements should employees contribute, and what drives their decisions to limit workplace contributions? Understanding Quiet Quitting is crucial because organizations increasingly depend on employees’ voluntary extra-role contributions to maintain competitive advantage, while employees increasingly question the personal utility of such discretionary effort. This tension makes Quiet Quitting not merely a temporary trend but a manifestation of evolving workplace dynamics that challenges traditional assumptions about employee engagement and organizational commitment.

Quiet Quitting stands in contrast with other, relatively well-understood problematic indifference-, withdrawal- and even explicitly harmful workplace behaviors, such as counter-productive work behaviors [28–30] defined as “intentional employee behavior that is harmful to the legitimate interests of an organization” [31] (p. 1241), voluntary turnover [32,33] defined as “…instances wherein management agrees that the employee had the physical opportunity to continue employment with the company, at the time of termination” [34] (p. 50), or burnout [35] defined as “...a state of exhaustion in which one is cynical about the value of one’s occupation and doubtful of one’s capacity to perform” [36] (p. 20). From depictions appearing in the literature [25] and popular media, Quiet Quitting broadly encompasses employees’ execution of their work-related tasks at a minimum level of compliance, coupled with feelings of comfort – even a principled right – to engage at work with this minimal level of workplace contribution [37,38].

The phenomenological origin of the Quiet Quitting construct conceptually is rooted in research that has focused on the development of a more comprehensive understanding of employees’ ongoing decisions to limit their work-related contributions, or even deliberately engage in deleterious workplace behaviors. This focus includes, for example, counter-productive work behavior from the organizational behavior domain [28,39], turnover intentions [40,41] and continuance commitment from the human resource management domain [42,43], burnout [35], and fatigue from the health psychology domain [44,45].

Since then, research on these kinds of dissociative employee behaviors has been linked to a wide range of negative outcomes, including for example, diminished overall job performance and organizational citizenship behaviors [46], depression [47], and general negative health consequences for employees [48]. The popular press has recently highlighted the growing incidence of Quiet Quitting, noting many of these detrimental outcomes [49–51]. Researchers and practitioners alike have begun to take note of the detrimental impacts of dissociative employee behaviors such as Quiet Quitting on employers’ bottom line outcomes, catalyzing growing popular and academic focus on the phenomenon.

In light of voluminous – and growing – theoretical and empirical evidence bearing on the importance of employee contributions extending above and beyond extreme minimum levels of workplace compliance [25,52,53], coupled with venerable recognition of the fragility of organizations in which employees provide strictly contractually adherent contributions [54,55], it is critical to develop a more comprehensive understanding of the Quiet Quitting construct.

Our theoretical understanding of Quiet Quitting is limited, emerging primarily from anecdotal and descriptive accounts in the popular and practitioner press [25]. In particular, theories and research bearing on employees’ decision to provide extreme minimum levels of workplace contribution have not coalesced around an agreed-upon conceptual definition of the phenomenon. This research also does not explain the mechanisms associated with the emergence of Quiet Quitting, which, of course, is important because understanding the drivers of this pattern of employee extreme minimum contribution positions employers to react and address this deleterious phenomenon. In line with these trends, there has been a dramatic increase in attention paid to these kinds of behaviors over the last decade [56–60]. Despite growing interest, the organizations literature lacks consensus on how Quiet Quitting should be conceptualized and measured.

In the current study, we define Quiet Quitting as work engagement at a minimum level of contribution, at a level that just – but only just – avoids involuntary turnover; coupled with feelings of comfort and personal satisfaction with this minimum level of work engagement A construct-valid, multidimensional measure of Quiet Quitting would accelerate development of theory and research in this domain by facilitating examination of how various dimensions of Quiet Quitting relate to conceptual correlates and outcomes.

This kind of systematic examination of the Quiet Quitting construct and its correlates is not currently possible, absent an empirically validated measure of the construct, with demonstrated differentiation from other related constructs and established psychometric validity.

In explicating the conceptualization of Quiet Quitting, it is also critical to establish the boundaries of the domain. As Podsakoff et al. [61] noted, “One reason why a measure/ manipulation may be contaminated is that the definition of the theoretical concept is ambiguous, too broadly defined, or lacks adequate precision” (p. 167). As such, the goals of this study are to (a) provide an overview of prior conceptualizations and measures of various forms of workplace behavior reflective of diminished or limited contribution, (b) provide a multidimensional conceptualization of Quiet Quitting which is conceptually distinguished and does not overlap with other related constructs, and (c) develop and validate a multidimensional measure of Quiet Quitting. The proposed two-factor structure of the Multidimensional Quiet Quitting Scale (MQQS) is an essential and critical tool to develop a professional understanding of the Quiet Quitting nomological network, and a more nuanced understanding of how the dimensions of Quiet Quitting relate to conceptually adjacent constructs and correlates, such as commitment. Before moving to the description of the scale development process, we begin with a review of existing conceptualizations and measurements of various forms of diminished employee work contribution and offer descriptions of their conceptual relationships with Quiet Quitting.

We organize the remainder of this paper as follows. First, we review existing conceptualizations and measurements of workplace behaviors reflecting diminished contribution, establishing the theoretical foundation for Quiet Quitting. We then present four complementary studies that systematically develop and validate the Multidimensional Quiet Quitting Scale (MQQS). Study 1 employs a deductive approach with MBA students to develop and refine scale items through content validation. Study 2 tests the scale’s initial psychometric properties and nomological validity using a Prolific sample of full-time employees. Study 3 extends validation to a global context through a worldwide snowball sample, while Study 4 provides a comprehensive test of the scale using Portuguese employee data with organizational controls. Together, these studies establish the MQQS as a reliable and valid measure of Quiet Quitting across different contexts and settings. We conclude by discussing theoretical and practical implications, limitations, and future research directions.

## Literature review

### Clarifying the conceptualization and measurement of quiet quitting

In order to establish a conceptualization to facilitate measurement of Quiet Quitting, we assembled adjacent construct definitions from research exploring various forms of diminished employee connection to work [62–65]. To advance this process, we also examined conceptualizations appearing in the practitioner and popular press [20–22,25]. Following recommendations offered by Podsakoff et al. [61], we closely evaluated these conceptualizations to determine key construct attributes as well as elements of the construct amenable to refinement.

In Table 1 we outline depictions of conceptually adjacent forms of diminished employee connection to work and examine overlapping and distinctive aspects of these constructs. One common attribute that these various depictions of diminished employee contribution share is that they reflect an employee-driven decision to modify work contributions or cognitions/affect associated with the modification of work contributions, rather than an organization-driven decision to modify an employee’s contributions. This attribute of these forms of diminished employee contribution to work coincides with the current conceptualization of Quiet Quitting, where employees make the explicit decision to engage with work and execute responsibilities on the job at an extreme-minimum level.

**Table 1:** Summary of conceptual correlates and measures of employees’ diminished connection with work.

Study	Conceptualization of diminished connection with work	Key attributes	Distinction between conceptualizations	Corresponding measure	Primary Critique
**Cammann et al. (1979)** [62]	**Turnover Intentions** is defined as “a conscious and deliberate willfulness to leave the organization.” [59], that emerges as “the last in a sequence of withdrawal cognitions, a set to which thinking of quitting and intent to search for alternative employment also belong	• future oriented • frequent operational/practical considerations related to leaving • future orientation/ tactical considerations relating to the search different work • affective/disappointment with current work situation • affective/retrospective disappointment with work choice	• present orientation • no considerations of actually leaving the job, just doing less work while on the job • no considerations of finding different work • explicit efforts to reduce disappointments with current work (QQ) • no retrospective reflections of work choice	Cammann et al. (1979) [62] – Turnover Intentions • thinking of leaving • looking • retrospective choosing	Reflects employees’ future inclination/plan to leave the job/ organization while QQ reflects current actions while remaining in the job/with the organization, providing an extreme minimal level of contribution while doing so. While TI is ‘leave’ oriented as a consequence of disappointment with current situation, QQ is ‘stay’ oriented as a consequence of creating a more satisfying situation
**Meyer et al. (1993)** [66]	**Continuance commitment** – “ a psychological state that (a) characterizes the employee’s relationship with the organization and (b) has implications for the decision to continue or discontinue membership in the organization.” [57]. Continuance commitment develops based on “the magnitude and/or number of investments (or side-bets) individuals make and a perceived lack of alternatives.” [67]	• present-oriented • practical/tactical recognition of being stuck or mired in the job • practical/tactical recognition of difficulty in changing current situation • affective - no feelings of loyalty to the organization • affective concerns over life disruption associated with leaving • practical/tactical recognition of having sunk costs • practical/tactical concerns over lack of alternatives	• no sense of being stuck in the job • no consideration of difficulties associated with alternative work • both CC and QQ reflect minimal loyalty to the organization • both CC and QQ encompass disruption, but CC encompasses disruptions over leaving while QQ encompasses disruptions over doing more than a minimum level of work	Meyer et al. (1993) [66] – Continuance Commitment • necessity • difficulty • disruption • options • investment • alternatives	Reflects frustration at being mired in a job as a consequence of historical levels of investment/current lack of opportunities, while QQ reflects actions employees take to diminish frustration with current work situation. CC explicitly reflects ‘leaving inhibitors’ while QQ explicitly reflect ‘staying facilitators’.
**Robinson & Morrison (2000)** [64]	**Psychological Contract Breach** is defined as “a subjective experience, referring to one’s perception that another has failed to fulfill adequately the promised obligations of the psychological contract” [64]	• present-oriented • practical/tactical recognition of having been deceived by employer • practical/tactical recognition of lack-of-follow-through on promises made • practical/tactical recognition of employer failing to provide material support • practical/tactical recognition of exchange violation	• both orbit levels of contribution • while Breach reflects org. violation of minimum exchange levels, QQ reflects employee violation of minimum exchange levels • violation reflects tactical consequences of unmet exchange by org. while QQ reflects tactical consequences of unmet exchange by employee	Morrison and Robinson (2000) [64] – Psychological Contract Breach • extent of unmet promises over time • trustworthiness • extent of unmet promises current • receipt of promised exchange • broken promises	Reflects employees’ feelings that their exchange stipulations with their organization have not been met while QQ reflects employees’ decision to provide an extreme-minimum level of contribution toward meeting their exchange obligation with their organization
**Robinson and Morrison (2000)** [64]	**Feeling of violation** Morrison and Robinson [68] describe a conceptual distinction between “perceived contract breach, which is a perception, and the experience of `contract violation’ which is an affective or emotional state that may or may not accompany that perception.” [64] (p. 532)	• present-oriented • extreme negative affect toward the organization • extreme negative affect/betrayal relating to the organization’s actions • practical/tactical – feelings of obligations not having been met • negative/affective resentment about treatment	• both reflect emotional consequences associated with exchange violations – while breach reflects employees’ negative reaction to org. violation, QQ reflects employees negative/positive reaction to contributing more/less than absolutely necessary	Robinson and Morrison (2000) [64] – Violation • anger • betrayal • violation • frustration	Reflects employees’ negative feelings in reaction to the perception that their organization has failed to meet its exchange obligations, while QQ reflects employees’ negative feelings in response to providing more than an extreme-minimum level of effort toward meetings their exchange obligations with their organization
**Maslach et al. (2001)** [35]; **Schaufeli et al. (1996)** [69]	**Cynicism** is defined as “an indifferent or distant attitude toward work in general and the people with whom one works, losing one’s interest in work and feeling that work has lost its meaning” [70] (p. 498)	• present-oriented attitude toward work • cognitive/evaluative response to conditions • psychological distancing from work role • loss of work meaning • indifference toward work relationships • emotional exhaustion not necessary	• while cynicism reflects psychological distancing, QQ reflects behavioral minimum contribution • cynicism emerges from resource depletion, QQ from deliberate choice • cynicism includes broad work alienation, QQ maintains minimal task engagement • cynicism affects work relationships, QQ focuses on task contribution • QQ can occur without emotional exhaustion or meaning loss	Maslach Burnout Inventory (1997) [36] - Cynicism subscale: • detachment • loss of enthusiasm • work meaninglessness • indifference	While both cynicism and QQ involve withdrawal, cynicism represents an erosion of psychological connection to work, while QQ represents a deliberate calibration of effort to minimum levels. Cynicism may lead to QQ behaviors, but QQ can occur without cynical attitudes.

For example, turnover intentions encompass plans by the employee to leave his/her current job and organization; conceptually, the most extreme form of diminished levels of contribution at work. The construct reflects an employee-driven anticipated extreme downward shift in current levels of work contribution. Also reflecting cognitions related to changing levels of contribution, continuance commitment encompasses anticipated life disruptions associated with the decision to potentially modify or reduce current levels of work contribution. Again, both constructs reflect an employee-driven shift in anticipated or potential contributions at work, rather than an employer-driven nexus for the anticipated or contemplated shift in contribution levels. Psychological contract breach also encompasses employees’ cognitions associated with current levels of exchange-related contributions, and reactions to understood levels of contribution. These conceptually adjacent forms of diminished employee contribution at work all share this attribute of being employee-driven.

A second common attribute these depictions of diminished employee contribution all share is that they have a component of negative affective valence. This attribute also coincides with the current conceptualization of Quiet Quitting, where employees have negatively valenced reactions associated with work contributions extending beyond an extreme-minimum level. For example, psychological contract breach encompasses employees’ evaluation of their organization’s failure to effectively execute on their (the employer’s) end of the understood exchange obligation at a minimally acceptable level, coupled with feelings of having been deceived as regards follow-through on these obligations on the part of the organization. These negative reactions explicitly orbit the organization’s extreme minimum exchange contributions, and employees’ recognition of this level of organizational failure to follow through on expected or anticipated obligations. Also reflecting employees’ negative reactions to the organization’s level of contribution, Violation encompasses employees’ feelings of resentment and betrayal at a perceived breach of the organization’s exchange obligation at a minimally acceptable level. Likewise, Turnover Intentions encompass employees’ disappointment with their current work situation and choice of work, ultimately anticipatory of the decision to voluntarily diminish current levels of contribution to an extreme minimum level. These forms of diminished employee contribution all share this negative valence in common.

While these depictions of diminished employee contribution share attributes in common, they also have some key conceptual distinctions from the Quiet Quitting construct. For example, while Turnover Intentions (TI) is future-oriented, reflecting plans to leave the job/organization – again, and also reflecting an extreme/ultimate downward shift relative to current levels of work contribution, Quiet Quitting (QQ) is present-oriented, reflecting current levels of work-related contribution. QQ also does not encompass considerations of actually leaving the job (a terminal level of diminished work contribution), while this cognition of terminally diminished contribution is central to the TI construct. QQ also does not encompass an anticipation of seeking different work or officially changing current work status, while this future-oriented, planning is a core element of the TI construct. While QQ does explicitly encompasses a reduction in current levels of work contributing to an extreme-minimum level, this diminished in-role contribution is not encompassed by the TI construct, which rather encompasses the anticipated termination of all work-related contributions. QQ also does not encompass retrospective consideration of work choice, being present-oriented, while this element of reflection is a key aspect of the TI construct.

Quiet Quitting also is conceptually distinct from Continuance Commitment (CC) in several ways. While QQ does not encompass the idea of feeling stuck with or mired in a particular role, or reflections relating to the difficulties associated with finding alternative work, these comparative attributes (i.e., feeling mired; difficulties associated with change) are central to the CC construct. In contrast, rather than feeling stuck or mired, QQ encompasses diminished work contributions, which are likely to diminish feelings of being stuck or mired in a role– given the explicit downward recalibration in work levels [71]. While both the CC and QQ constructs reflect a minimal level of loyalty to the organization, and both encompass the notion of personal disruption or difficulty, CC emphasizes anticipated personal disruptions associated with the decision to leave the job, while QQ emphasizes anticipated personal disruption associated with the decision to engage at work beyond an extreme-minimum level of contribution. Thus, while CC explicitly encompasses the notion of personal disruption associated with leaving, QQ explicitly encompasses the notion of personal disruption associated with staying.

QQ also shares some commonalities – but also key distinctions from both Psychological Contract Breach (PCB) and Violation as well. For example, all three constructs are present-oriented (as opposed to being future-oriented as in the case of TI). However, while PCB encompasses employees’ perception that the organization has violated key aspects of the implied exchange relationship, and Violation encompasses employees’ affective reactions to these violations, QQ encompasses employees’ decision to execute their exchange responsibilities at an extreme-minimum level. A key distinction between PCB/Violation and QQ is that while the former encompasses employees’ evaluations/reactions to the organization’s extreme-minimum (i.e., insufficient) level of exchange contributions, the latter encompasses employees’ extreme-minimum (i.e., insufficient) level of exchange contributions. Both constructs encompass the notion of exchange insufficiency, but while PCB and Violation reflect insufficiency that emerges from the institutional partner in the exchange, QQ reflects insufficiency that emerges from the individual partner in the exchange.

What emerges from our review of conceptually adjacent constructs reflecting diminished connection with work are attributes that we integrate into our multidimensional conceptualization of Quiet Quitting. There were some attributes of these constructs that we did not choose to incorporate into our conceptualization, such as leaving or planning to leave the organization, consideration of the life disruptions associated with leaving, or the organization’s efforts to satisfy exchange obligations. We also did not include the organization’s current levels of exchange contribution in their exchange with employees or employees’ reactions to their organization’s current level of exchange contribution. These elements were excluded from the conceptualization of Quiet Quitting to diminish potential conceptual boundary overlaps between Quiet Quitting and other conceptually adjacent or related constructs reflective of diminished work connection.

### A multi-dimensional quiet quitting conceptualization

We now move to a discussion of the attributes of Quiet Quitting, which defines the overall Quiet Quitting construct; each representing a necessary but in isolation insufficient reflection of Quiet Quitting. For example, while engaging at work at an extreme-minimum level of contribution is a central aspect of Quiet Quitting, simply doing as little work as possible while on the job (i.e., to avoid involuntary turnover) – alone – does not amply embody the domain of the construct. Quiet Quitting also encompasses an affective element as well, reflecting the affective experience associated with engaging at work at an extreme-minimum level of contribution.

While the emotional component of quiet quitting could theoretically be subdivided into distinct negative reactions to over-contribution and positive reactions to minimal contribution, we propose a unified emotional dimension for several reasons. First, social exchange theory suggests that emotional reactions to exchange relationships tend to be reciprocal - negative affect toward perceived over-contribution typically corresponds with positive affect toward maintaining exchange equilibrium [71]. Second, our empirical evidence across multiple samples consistently supports a two-factor structure where emotional reactions, both positive and negative, load together reliably. However, we acknowledge that in certain contexts, such as situations of work-home interference or role stress, employees might experience strong negative reactions to extra work without corresponding positive feelings about minimal contribution. Future research might productively explore conditions under which these emotional responses dissociate.

From our review, it emerges that Quiet Quitting is a multidimensional construct that falls within a nomological network of distinct but related constructs. In the current study, we define Quiet Quitting as a pattern of workplace behavior characterized by (1) deliberate restriction of work-related effort to a minimum level that just avoids involuntary turnover, coupled with (2) feelings of comfort and personal satisfaction with this minimal level of work contribution. This definition distinguishes Quiet Quitting from the established construct of work engagement [72, 73], which represents a positive, fulfilling, work-related state of mind. While work engagement captures an employee’s vigor, dedication, and absorption in their work role, Quiet Quitting represents an intentional withdrawal of effort to minimal levels and associated satisfaction with such minimal contribution. This conceptual clarity is important given that Quiet Quitting may represent the behavioral manifestation of severely diminished work engagement, though the relationship between these constructs remains an empirical question for future research.

**Behavioral Quiet Quitting** – an extreme minimum level of workplace contribution at a level just above normative criteria bearing on involuntary turnover. First, quiet quitting encompasses a profile of workplace behavior that just – but barely – satisfies extreme minimum standards of understood explicit or implicit exchange contribution with the organization [38]. Virtually all of the conceptually adjacent constructs appearing in Table 1 encompass levels of contribution or exchange, with scholars reflecting on employees’ anticipated future behavioral response (e.g., terminal decrease in contributions) [62], employees’ cognitive reactions (e.g., perceptions of being mired or stuck in a role) [63], and affective reactions (e.g., extreme negative reactions/feelings of betrayal) [64].

Important to our conceptualization is the recognition that emotional experiences and cognitive evaluations are not merely consequences of Quiet Quitting behavior, but rather constitute integral components of the phenomenon itself. While items reflecting perceptions of burnout, mental health, and evaluations of minimal work behavior might appear to capture outcomes, we argue these represent concurrent emotional-evaluative experiences that are fundamental to the Quiet Quitting construct. This follows established precedent in construct development where emotional experiences and their cognitive appraisals are treated as constituent elements rather than consequences (e.g., job satisfaction’s affective and cognitive components; [74]). The simultaneous nature of behavioral restriction and emotional-evaluative experiences in Quiet Quitting distinguishes our conceptualization from sequential models where behaviors lead to separate emotional consequences. The integration of behavior and concurrent emotional-cognitive experiences provides a more complete representation of Quiet Quitting as it manifests in organizational settings.

The behavioral dimension of Quiet Quitting is rooted in social exchange theory (SET) [71,75]. According to SET, people apply economic principles in the evaluation of their relationships, comparisons of alternative partnerships, and their levels of contribution to the exchange. As economic actors, people try to maximize the rewards potentially available through these relationships and minimize the costs of these relationships [76]. People will tend to remain in relationships where the benefits of doing so are perceived to exceed the relationship’s costs. SET reflects on the levels of contribution that people are willing to make to an exchange, and the reactions that tend to emerge when perceived contributions to the exchange exceed or lag the perceived benefits of the exchange [68,71].

In exchange relationships where people perceive the benefits they receive from the relationship exceed their current levels of contribution, they can feel emotional tension that compels them to increase their levels of contribution to the exchange [52,53,55]. In the organizational behavior domain, this tension is theorized to lead to a broad range of enhanced work-related contributions, including organizational citizenship behaviors (OCB). In contrast, when the perceived costs associated with an exchange exceed the relationship’s perceived benefits, individuals have several options available to them, which can include diminishing their current levels of contribution to the exchange [77,78] or leaving the exchange altogether [79].

When the costs associated with leaving the organization are perceived to be too high, in anticipation of deficient available alternatives [63], an option that SET predicts in reaction to exchange contributions over perceived benefits of the exchange is that employees are likely to decrease their levels of contribution, reflected in behavioral Quiet Quitting. With behavioral Quiet Quitting employees engage in a minimum level of contribution, at a level that just – but only just – avoids involuntary turnover.

**Emotional Quiet Quitting** – affective response to doing more than is required. Second, Quiet Quitting encompasses a profile of extreme negative emotional reaction to providing work contributions exceeding an extreme minimum standard of exchange [38]. Again, virtually all of the conceptually adjacent constructs depicted in Table 1 encompass emotional reactions in response to levels of contribution or exchange, with scholars reflecting on employees’ disappointment with current work conditions (e.g., retrospective disappointment) [62], employees’ cognitive reactions (e.g., concerns over life disruption) [63], and feelings of violation (e.g., betrayal) [64]. As with the behavioral dimension of Quiet Quitting, emotional Quiet Quitting also is rooted in social exchange theory. Central to SET is the emergence of negative emotions in response to the perception that feelings of contribution to the exchange exceed the exchange’s inherent benefits [e.g., 80]. SET provides that these negative feelings are likely to focus directly on the contributions being offered within the exchange. With emotional Quiet Quitting employees feel a heightened level of negative emotion associated with work-related engagement beyond an extreme minimum level of work contribution and a heightened level of positive emotion associated with work-related engagement at, or below, this extreme minimum level of contribution.

### Validation strategy

There are several steps associated with the validation of a measure [81], the first of which focuses on generating evidence that: the items designed to capture the domain of the construct reflect the content domain (i.e., content validity), the measure is psychometrically sound (e.g., internal consistency; expected dimensionality), and that the measure has the expected associations with constructs within its nomological network (i.e., convergent, discriminant, criterion). We first establish the content validity of the Quiet Quitting measure, then establish the measure’s psychometric properties, and finally evaluate the measure’s construct validity. Table 2 provides a summary of analyses conducted, and samples leveraged in this process. Because the steps in this process used different samples, we present the logic for each hypothesis and follow this logic by describing all of the samples and scales used in these studies.

**Table 2:** Summary of research design, samples, variables, measures and methods.

Study	Design & purpose	Sample size	Variables & measures	Methods
Study 1: Item Generation and Content Validity of the MQQS	Research Design: (1) item generation (SMEs—two authors) and (2) content validity assessment (SMEs—MBA students) Purpose: Initial scale development and content validation through structured item-sort task Design Elements: - Randomized presentation of definitions - 7-point Likert ratings - Two-round rating process	37 MBA students	• Scale items for QQ Behavioral and QQ Emotional • Expert ratings of item-dimension correspondence	• One-way ANOVA for content adequacy • Item retention based on statistical significance and theoretical relevance
Study 2: Psychometric Properties of the MQQS	Research Design: Cross-sectional survey validation study Purpose: Initial scale validation using working adults Design Elements: - Online survey platform - Full-time employee screening - Comprehensive nomological network testing	985 full-time employees	• QQ dimensions • Voice • Affective, Continuance, & Normative Commitment • Contract breach & violation • Social desirability • OCBI, OCBO • Turnover intentions	• Loevinger’s coefficient • CFA with SRMR/R² validation • OLS regression • Relative weights analysis
Study 3: Cross-Validation of the MQQS in Diverse National/Cultural Settings	Research Design: Multi-country cross-sectional validation study Purpose: Cross-cultural validation and replication Design Elements: - Social media recruitment - Newsletter distribution - Multi-channel sampling approach	2,128 employees worldwide	Same variable set as Study 2	Same analytical approach as Study 2, with additional tests for measurement invariance
Study 4: Cross-Validation of the MQQS in a Field Setting	Research Design: Organization-embedded field study Purpose: Validation with organizational context and controls Design Elements: - Linked employee-organization data - Industry-clustered sampling - Archival financial measures	1,897 full-time employees	Same variables as Studies 2-3, plus: • Employee demographics • Organizational characteristics • Financial performance metrics • Industry controls	Same analyses as Studies 2-3, plus: • Industry-clustered standard errors • Multi-level modeling • Organizational control variables

### Hypothesis development

#### Psychometric properties.

The MQQS was designed to capture two conceptually related dimensions of the Quiet Quitting construct, behavioral and emotional Quiet Quitting. Because we aimed to establish the viability of this two-dimensional structure, it was critical to substantiate and replicate the measure’s factor structure using a number of different samples.

#### Nomological network validity.

The convergent and discriminant validity of the MQQS was tested by evaluating comparisons with a number of conceptually adjacent constructs from the nomological network in which Quiet Quitting is embedded. Approaching this evaluation with a focus on providing a conservative test of MQQS’s nomological validity, we included correlates with definitions widely accepted from the literature, and which also have been repeatedly evaluated using well-cited, commonly referenced, and repeatedly psychometrically validated scales [82]. To establish convergent validity, we included constructs for comparison that have demonstrated both conceptual and empirical associations with what we conceptualize as extreme minimum levels of workplace contribution.

Three psychological states were included in this evaluation: affective, normative, and continuance commitment, e.g., [63]. Commitment is reflective of a psychological state that both characterizes the nature of the subjective relationship an employee has with their employer, and also bears directly on the cognitions employees develop regarding their intentions to remain with their organization. These forms of commitment reflect very different underlying motivations. While employees with affective commitment tend to remain in their role with their organizations because they want to remain, employees with continuance commitment tend to remain because they perceive little value in their potentially available alternatives, while employees with normative commitment remain because of feelings of responsibility to the organization to do so [63,83].

Consistent with these conceptual distinctions, while affective and normative commitment tend to be negatively associated with negatively valenced work experiences such as role conflict, and role ambiguity [46], these forms of commitment also tend to be positively associated with positively valenced experiences such as job satisfaction, task performance [84,85] and interactional, procedural and distributive justice [46]. In contrast, while affective and normative commitment tend to have positive associations with positively valenced employee work experiences, continuance commitment tends to be correlated with negatively valenced constructs. For example, continuance commitment tends to be positively related to various forms of role stress, such as role conflict and ambiguity [46], and negatively related to various forms of justice such as procedural, distributive, and interactional justice [46].

Two psychological contribution-centric perceptual correlates also were examined, psychological contract breach and psychological contract violation [37,64]. While psychological contract breach is defined as a subjective experience bearing on an employer’s failure to meet their explicit or implicit obligations [37], psychological contract violation is defined as the affective or emotional reaction an employee experiences following recognition of a psychological contract breach. Thus, while both violation and breach emerge as a consequence of employees’ perceptions of distance between implied/promised employer contributions and experienced contributions, while breach encompasses awareness, violation encompasses reactions [68].

Consistent with these conceptual definitions, psychological contract breach tends to be negatively related to a range of positively valenced correlates, and negatively related to a range of positively valenced correlates. For example, psychological contract breach is negatively related to experienced job satisfaction, organizational citizenship behaviors, and in-role performance [86,87], and positively related to employee mistrust, voluntary turnover, and feelings of violation [87]. Similar relationships tend to emerge for violation, which is negatively related to justice perceptions, performance, and job satisfaction [64,88,89]. Finally, we also included one behavioral correlate of Quiet Quitting, reflective of employees’ focus on making improvements to their work unit and organization - employee voice [90,91]. Quiet quitting reflects an extreme minimum level of workplace contribution, coupled with positive feelings associated with this level of contribution. Voice behavior, in contrast, reflects employees’ efforts to actively improve the functioning and operation of their places of work, even to the point of putting themselves at potential social risk for doing so [92]. In contrast with Quiet Quitting, voice behaviors tend to be associated with work engagement [93] and commitment to the organization [94]. Thus, based on evidence bearing on theses pattern of relationships, we propose the following:

Hypothesis 1: MQQS presents a distinct nomological net in relation to: 1) continuance commitment, 2) perceived contract breach, and 3) perceived contract violation, and is negatively related to 4) employee voice behavior and 5) affective commitment.

#### Criterion-related validity.

To establish the managerial relevance of the quiet quitting construct, it is critical to demonstrate that MQQS is associated with important outcomes in ways that coincide with the construct’s theoretical underpinnings. While Quiet Quitting broadly encompasses employees’ execution of their work-related tasks at a minimum level of compliance, coupled with feelings of comfort with this minimal level of workplace contribution, turnover intentions represent a distinct future-oriented withdrawal cognition that may emerge as a consequence of sustained Quiet Quitting behavior. This temporal sequence aligns with established models of workplace withdrawal, where immediate behavioral and emotional responses to workplace conditions often precede the development of turnover intentions [65]. Positioning turnover intentions as a criterion variable allows us to test whether Quiet Quitting, as a form of “internal withdrawal,” may lead to considerations of “external withdrawal” through turnover - an important practical consideration for organizations.

To establish the managerial relevance of the Quiet Quitting construct, it is critical to demonstrate that MQQS is associated with important outcomes in ways that coincide with the construct’s theoretical underpinnings. From this vantage point, we focused particularly on organizational citizenship behaviors (OCBs) as a form of extra-role performance that extends beyond employees’ formally prescribed job duties [54,95]. While quiet quitting reflects minimal engagement in required in-role behaviors, OCBs represent discretionary behaviors that exceed formal role requirements and contribute to organizational effectiveness [52,55]. Building on social exchange theory [71] and evidence that extra-role performance emerges from employees’ willingness to “go the extra mile” beyond conventional contribution levels [96,97], we expect both Behavioral and Emotional QQ to be negatively related to OCBs. Because employees engaged in Quiet Quitting deliberately restrict their efforts to minimal in-role requirements and feel positively about avoiding additional work contributions, they are particularly unlikely to engage in discretionary extra-role behaviors that characterize citizenship performance. This theoretical positioning of OCBs as an outcome provides a stringent test of Quiet Quitting’s implications for behaviors that, while not formally required, are crucial for organizational effectiveness. Because employees engaged in QQ both do as little work as possible to avoid being fired, and also feel positive about doing as little work as it is possible for them to do to avoid sanction, they are unlikely to engage in behaviors that reflect significant variation beyond status-quo expectations. This positioning of turnover intentions as a criterion variable, alongside OCBs, provides a more theoretically coherent framework for understanding how Quiet Quitting may influence important organizational outcomes.

Finally, in light of the work-focused content of the MQQS construct, we assessed the relationship between MQQS and employees’ expected future work-related contributions; turnover intentions. Turnover intentions reflect employees’ conscious willfulness to leave the organization – i.e., an extreme minimum level of contribution [65], and which emerge as among the final cognitions employees has as plans develop to seek alternative employment. Because employees engaged in Quiet Quitting actively do as little work while on the job as possible, and feel emotional satisfaction associated with their decision to withhold relevant work-related efforts, this pattern reflects a consistent absence of loyalty to the organization or its goals. The expectation that the pattern of work-related behavior reflective of Quiet Quitting is likely to be positively associated with turnover intentions coincides with meta-analytic evidence of negative association between variables reflective of on-the-job embeddedness and turnover intentions [98]. Thus, we also propose the following:

Hypothesis 2: MQQS is negatively related to OCB-I, OCB-O, and positively related to turnover intentions

## Methods and results

### Study 1—item generation and content validation of the MQQS

#### Item generation.

We used a deductive approach to create items for the MQQS, following established procedures recommended by Hinkin [81] and Schwab [99]. To ensure the items were clear, consistent, and not confusing, we followed Hinkin’s [81] recommendations and created items that were simple to understand and not double-barreled. This process resulted in the creation of 22 items, which we then screened for representativeness and redundancy, as recommended by previous scale development researchers [100]. Any items deemed redundant or nonrepresentative were eliminated from the original set of 22 items, leaving a total of 20 unique items that were designed to measure MQQS (10 items for behavioral Quiet Quitting and 10 items for emotional Quiet Quitting).

#### Content validation.

To assess the content validity of the initial set of MQQS items, following established procedures [101], an item-sort task was conducted [82,100]. The item-sort task involves “the deletion of items that are deemed to be conceptually inconsistent” [81] (p. 108). To perform the task, we adopted Hinkin and Tracey’s [101] one-way analysis of variance (ANOVA) procedure. This procedure directly assesses an item’s content validity “by comparing the item’s mean rating on one conceptual dimension to the item’s ratings on another comparative dimension” [101] (p. 181). By using the one-way ANOVA approach, we were able to compare the mean ratings for each scale item on both the behavioral and emotional dimensions of the scale.

#### Participants and procedure.

We recruited 37 MBA students with little professional work experience and administered an item-sort task online using a Qualtrics survey. The definitions for behavioral Quiet Quitting and emotional Quiet Quitting were presented to participants in a random order, and participants rated the extent to which each of the 20 initial items aligned with the definitions for each of the two dimensions of the MQQS (10 items per dimension). Ratings were provided using a seven-point Likert-type scale ranging from 1 (strongly disagree) to 7 (strongly agree). Participants completed two sets of ratings, one for behavioral Quiet Quitting and one for emotional Quiet Quitting, with each item being rated twice.

#### Results.

As can be seen in Table 3, the results from the one-way ANOVA indicated that 8 of the initial set of 20 items were rated significantly higher (*p* < .05) on their intended dimension than on their unintended dimension (i.e., a total of 3 behavioral quiet quitting items and 5 emotional quiet quitting items), leading to the elimination of 7 items. While some items in our initial set were eliminated due to their lack of statistical significance across dimensions, we elected to retain five items that were both consistent with our original expectations and that also were consistent with the expected direction of the relationship. We based this decision on the effect sizes associated with these items, in conjunction with their substantive importance for capturing different aspects of the construct being measured [82]. In particular, we found that these items were able to distinguish important nuances in the dimensions of the construct that were not captured by the other items in the scale. This iterative item-culling process resulted in a final set of 13 items, among which were retained 6 items reflecting behavioral Quiet Quitting and 7 items reflecting emotional Quiet Quitting.

**Table 3:** Results of content adequacy analysis (one-way ANOVA).

Item	Behavioral	Emotional
**Behavioral items (B1-B10)**		
**1. I do only the work I’m specifically asked to do; just enough to not lose my job. (B1)**	**5.05 (2.27)**	**4.19 (1.88)**
**2. I spend the adequate time necessary working on tasks to keep my job. (B2)**	**5.27 (1.31)**	**4.54 (1.57)**
3. By doing just enough to keep my job, I am okay foregoing future advancement. (B3)	4.27 (2.14)	3.97 (1.89)
**4. I do not do extra work beyond what I’m paid to do. (B4)**	**5.00 (2.13)**	**4.16 (1.83)**
5. I prefer not to return emails/calls on holidays and vacations. (B5)	4.92 (1.72)	4.89 (1.81)
6. While increasing the odds of just keeping my job, I try to maintain a strict boundary between work and my life outside of work. (B6)	4.97 (1.74)	4.73 (1.82)
*7. I don’t look for extra work to do even though it could help me to get promoted. (B7)*	*4.65 (2.16)*	*4.08 (2.07)*
8. Unlike others, I take active steps at work to reduce the job as a part of my identity. (B8)	4.22 (1.58)	4.49 (1.84)
*9. To keep my job, I believe in working just enough, not harder or smarter. (B9)*	*4.81 (2.15)*	*4.19 (1.82)*
*10. Doing only the work that is required is smart, not lazy. (B10)*	*4.73 (1.82)*	*4.38 (1.91)*
**Emotional items (E11-E20)**		
11. It negatively affects my mood when I do more work for the same pay. (E1)	4.73 (1.76)	5.05 (1.89)
12. I feel guilty doing only the minimum amount of work necessary to keep my job. (E2)	3.49 (2.01)	3.86 (2.03)
*13. It doesn’t bother me when extra work gets left unfinished. (E3)*	*4.49 (2.00)*	*4.86 (1.84)*
**14. It is emotionally rewarding for me not looking for extra work to do. (E4)**	**4.16 (1.77)**	**4.89 (1.96)**
**15. Worries weigh heavily on my mind, when going above and beyond in my work. (E5)**	**3.59 (1.66)**	**4.95 (1.79)**
**16. My emotions often multiply negatively when working beyond what is necessary to keep my job. (E6)**	**3.89 (1.87)**	**4.81 (2.07)**
*17. I feel less burned out by doing only the work that is needed to keep my job. (E7)*	*4.54 (1.79)*	*5.19 (1.70)*
**18. My mental health is a lot better by not going the ‘extra mile’ at work. (E8)**	**4.14 (1.81)**	**5.14 (1.67)**
**19. I don’t tend to spend time mulling over work-related issues. (E9)**	**4.03 (1.88)**	**4.76 (1.77)**
20. Thinking about work issues only enough to keep my job makes my life outside work better. (E10)	4.46 (1.74)	4.78 (1.83)

Sample Size =  37.

Responses were made on a scale ranging from 1 (Strongly Disagree) to 7 (Strongly Agree).

B1-10: Behavioral QQ item, E11-20: Emotional QQ item. Standard deviations are in parentheses.

**Boldface** type denotes a significantly higher (p < .05) mean score with one-tailed test. 8 of the initial set of 20 items were rated significantly higher (p < .05) on their intended dimension than another dimension.

Items in *italicized* were retained for further analysis despite a lack of statistical significance across dimensions. 5 items were retained for further analysis despite a lack of statistical significance across dimensions. This decision was based on their alignment with our original expectations and directions, as well as their substantive importance for capturing different aspects of the construct being measured [102]. Effect sizes associated with these items were also taken into consideration.

### Analytical strategy for studies 2-4

For the scale validation studies (Studies 2–4), we use the following analytical approach. First, to identify the scale items that could potentially be excluded from the items identified in Study 1, we used Loevinger’s H coefficients. Originally proposed by Mokken [103], Loevinger’s coefficient, rather than depending on more subjective decision-making for culling items from the original item pool, this approach uses item parceling, and provides cutoffs based on the quality of all pairs of items in the scale:


$$H_{ij}=1-\frac{ObservedN_{ij}\left(1,0\right)}{ExpectedN_{ij}\left(1,0\right)}$$ (1)

The expected value is calculated based on the null hypothesis that items are independent of one another. If no errors are observed $H_{ij}=1$, and if as many errors as expected are observed under independence, then $H_{ij}=0$. Larger Loevinger coefficients indicate an improving combination of scale items. The cut-offs for $H_{ij}$are as follows: $H_{ij}<0.3$ indicates poor scalability; $H_{ij}<0.4$ indicates useful but weak scalability; $H_{ij}<0.5$ indicates acceptable scalability; and $H_{ij}>0.5$ indicates good scalability. For additional details, we refer readers to Hardouin et al. [104] who reflected on the utility of the item-parceling approach. In addition to using the Loevinger cutoffs, we also incorporated recommended CFA factor-loading cutoffs into our decision-making. In determining whether to retain a scale item, if $H_{ij}<0.3,$ and the CFA loading is poor (i.e., < 0.5), we dropped the item. if $H_{ij}>0.3.$ But, if the CFA loading was above the recommended cutoff of 0.5, we retained the item.

Second, for assessing the structure of the MQQS behavioral and emotional scales in CFA with the related scales in the nomological validity net (i.e., the constructs described under Study 2), we did not model any covariances among the scale items, or constructs that would artificially improve model fit. Recent research also has cautioned against the use of Chi-square values and fit indices as cut-off values. For example, Ximénez et al [105] argued that “holding model misspecification constant, the behavior of fit indices depends on factors such as the number of variables being modeled (model size), and the average observed correlation (magnitude off loadings or measurement quality). When a biased estimator of a fit index is used (e.g., CFI, TLI, or GFI), the behavior of the sample indices depends on sample size, rendering establishing cutoff values impossible. When an unbiased estimator is used (e.g., SRMR, or RMSEA), the behavior of the indices matches that of the focal population parameter and depends on the average *R*^*2*^ of the observed variables (communality); and for the RMSEA, also on the model size” (p. 368).

From Ximénez et al [105], who built on the approach reported by Shi et al. [92], who used a cutoff value for this index that is a function of SRMR divided by the average communality of the observed variables, *R*^*2*^, with “close fitting” models having SRMR/*R*^2^ ≤  0.05 and, “adequate fitting” models with SRMR/*R*^*2*^ ≤  0.5. However, based on added considerations of sample size, model misspecifications, and communality, Ximénez et al [105] presented the unbiased SRMR values in their Table 4 (p. 376). The communalities among the loadings of our scale items are high (i.e., the lowest was 0.58 for Study 3), and with 12 variables included in the CFA indicate, at worst, a model misspecification of 0.9 (i.e., with 1 as a model parameter identical to population parameters). We followed the approach reported by Ximénez et al [105], who suggested that

**Table 4:** Study 2- descriptives.

		Mean	SD	Min	Max	(1)	(2)	(3)	(4)	(5)	(6)	(7)	(8)	(9)	(10)	(11)
1	QQ Behavioral (without second item)	2.69	1.09	1.00	5.00	1.00										
2	QQ Emotional	2.94	0.97	1.00	5.00	0.80^*^	1.00									
3	OCBI	3.93	0.79	1.00	5.00	-0.55^*^	-0.47^*^	1.00								
4	OCBO	4.12	0.57	2.29	5.00	-0.44^*^	-0.38^*^	0.41^*^	1.00							
5	Turnover intention	2.58	1.24	1.00	5.00	0.38^*^	0.44^*^	-0.30^*^	-0.26^*^	1.00						
6	Voice	3.78	0.93	1.00	5.00	-0.44^*^	-0.38^*^	0.61^*^	0.32^*^	-0.29^*^	1.00					
7	Affective Commitment	3.12	1.18	1.00	5.00	-0.47^*^	-0.51^*^	0.45^*^	0.32^*^	-0.74^*^	0.45^*^	1.00				
8	Continuance Commitment	3.02	1.00	1.00	5.00	0.10^*^	0.15^*^	-0.09^*^	-0.12^*^	0.12^*^	-0.13^*^	-0.10^*^	1.00			
9	Normative Commitment	2.81	1.14	1.00	5.00	-0.44^*^	-0.47^*^	0.42^*^	0.24^*^	-0.60^*^	0.36^*^	0.78^*^	0.05	1.00		
10	Perceived contract breach	2.37	1.16	1.00	5.00	0.33^*^	0.41^*^	-0.29^*^	-0.267^*^	0.63^*^	-0.30^*^	-0.62^*^	0.19^*^	-0.51^*^	1.00	
11	Feelings of violation	1.89	1.14	1.00	5.00	0.31^*^	0.37^*^	-0.25^*^	-0.25^*^	0.63^*^	-0.29^*^	-0.57^*^	0.21^*^	-0.45^*^	0.83^*^	1.00
12	Social desirability	3.65	0.74	1.00	5.00	-0.22^*^	-0.23^*^	0.33^*^	0.36^*^	-0.19^*^	0.30^*^	0.26^*^	-0.09^*^	0.22^*^	-0.16^*^	-0.13^*^

*N*=985;

*p<0.05.

*“researchers should favor the use of the unbiased indices [and] not only a single cutoff for assessing the degree of fit of a model but different cutoffs based on the average value of the factor loadings. Our recommendation is to favor the use of the unbiased SRMR index of Maydeu-Olivares [* 106 *] with the correction proposed by Shi et al. [* 107 *] as a function of the communality (SRMR/R* ^*2*^ *) for the correct interpretation of the SRMR. Some years ago, Bentler [* 108 *] also offered this recommendation, to report the SRMR, arguing that SRMR needed little further research, as it is interpretable on its own.” (p. 378).*

Finally, our incremental validity test assessed whether the emotional and behavioral Quiet Quitting measures were redundant with the nomologically related constructs included in the analysis, and whether the measures explained unique variance in a set of several conceptually endogenous outcomes discussed in Hypothesis 2. For this test, we chose Voice; Affective Commitment; Continuance Commitment; Normative Commitment; Perceived contract breach; Feelings of violation; and Social desirability as co-predictors of variation in the model’s outcomes; and OCBI, OCBO, and turnover intentions as the outcomes that could be explained by variation in the MQQS. Although we expected similar relationships to emerge based on commitment type and contract breach perceptions, social desirability in the reporting of quiet quitting also may be salient. Our expectation was that MQQS would account for unique variance in these outcomes above any beyond that accounted for by the co-predictors in the model.

As noted by Clark et al. [109], using hierarchical multiple regression is problematic “when the predictors are highly correlated with one another [110,111], as is the case here with multiple measures and dimensions” in the proposed nomological net (p. 1296). Therefore, based on the caution reported by Clark et al. [109], we examined the unique variance that each predictor contributed to the total variance explained in the set out focal outcomes by conducting relative weights analyses (RWAs). The standardized relative weights can be interpreted as the percentage of unique variance explained by a construct.

For studies 2-4, we describe our sampling plan, all data exclusions (if any), all manipulations, and all measures used in the study. Analysis code and research materials are available at [OSF link not provided to preserve anonymity, but provided to the editor]. Data are not available publicly due to confidentiality. Data were analyzed using Stata 17 and the packages -validscale- and -domin-. The hypotheses and analysis were preregistered [OSF link shared with the editor]. The code for replication is available on the Open Science Framework site [the link is shared with the editor].

### Study 2. Psychometric properties of the MQQS

Ethics Commission approval for Study 2 was received from the institution of the 2nd author on September 19, 2022 (and then an addendum to the original approval on October 10, 2022, where we updated the consent letter) with the approval number 22/2022 (and 23/2022 for the addendum).

Participants in Study 2 were recruited from the Prolific survey panel on October 15, 2022. Individuals who were self-identified as full-time employees (i.e., at least 35 hours per week), and resided in the United States were eligible to participate in the survey. One thousand qualified participants completed the 10-minute survey and were compensated $1.80 for their participation. Of the 1000 participants who completed the survey, based on case-wise deletion, our final sample included 985 full-time employees. Measures included in our tests of nomological validity included OCBI, OCBO, Voice, Turnover intentions, Affective commitment, Continuance commitment, Perceived contract breach, Feelings of violation, and Social Desirability.

### Measures

#### Behavioral quiet quitting.

Participants rated six Behavioral Quiet Quitting scale items on a 5-point scale ranging from 1 (strongly disagree) to 5 (strongly agree). Unless otherwise noted, the measures included in the test of the nomological validity of the MQQS all were rated using the same 5-point scale. The following items were included in the measure: 1) “I do only the work I’m specifically asked to do; just enough to not lose my job”; 2) “I spend the adequate time necessary working on tasks to keep my job”; 3) “I don’t look for extra work to do even though it could help me to get promoted”; 4) “To keep my job, I believe in working just enough, not harder or smarter,” 5) “I don’t do extra work beyond what I’m paid to do” and 6) “Doing only the work that is required is smart, not lazy.” The reliability of the scale was α =  0.90 (the scale reliabilities for Study 2 are present in Table 8)

#### Emotional quiet quitting.

Participants rated the following seven Emotional Quiet Quitting scale item: 1) “It doesn’t bother me when extra work gets left unfinished,” 2) “It is emotionally rewarding for me not looking for extra work to do,” 3) “Worries weigh heavily on my mind, when going above and beyond in my work,” 4) “My emotions often multiply negatively when working beyond what is necessary to keep my job,” 5) “I feel less burned out by doing only the work that is needed to keep my job,” 6) “My mental health is a lot better by not going the ‘extra mile’ at work,” and 7) “I don’t tend to spend time mulling over work-related issues. The reliability of the scale was α =  0.87.

#### OCB-I.

OCB-I was measured using the OCB-I subscale of the Williams and Anderson’s [112] OCB measure. The OCB-I subscale consists of 7 items. Example items included “I help others who have been absent”; “I help others who have heavy work-loads”; “I assist my supervisor with his/her work (when not asked)”; and “I take time to listen to co-workers’ problems and worries”. The reliability of the scale was α =  0.86.

#### OCB-O.

OCB-O was measured using the OCB-O subscale of the Williams and Anderson [112] OCB measure. The OCB-O subscale also consists of 7 items, examples of which included “My attendance at work is above the norm”; “I give advance notice when I am unable to come to work”; “I take undeserved work breaks (reversed)”; and “I adhere to informal rules devised to maintain order”. The reliability of the scale was α =  0.67.

#### Voice behavior.

Voice behavior was measured using the 6-item measure developed by Van Dyne & LePine [113]. Example items included “I develop and make recommendations concerning issues that affect my work group”; “I speak up and encourage others in my group to get involved in issues that affect the group”; “I communicate my opinion about work issues to others in my group even if my opinion is different and others in the group disagree with me”; and “I keep well informed about issues where my opinion might be useful to my work group”. The reliability of the scale was α =  0.93.

#### Turnover intentions.

Turnover intentions were measured using the 3-item measure reported by Camman et al. [62]. Items included “I often think of leaving my organization”; “It is very possible that I will look for a new job next year”; and “If I could choose again, I would choose to work for my current organization (reversed)”. The reliability of the scale was α =  0.84.

#### Affective commitment.

Affective commitment was measured using the affective commitment subscale of the organizational commitment scale reported by Meyer et al. [63]. The affective commitment subscale consists of 6 items, examples of which included “I would be very happy to spend the rest of my career with my organization”; “I really feel as if my organization’s problems are my own”; “I do not feel a strong sense of “belonging” to my organization”; and “I do not feel “emotionally attached” to my organization”. The reliability of the scale was α =  0.93.

#### Continuance commitment.

Continuance commitment was measured using the continuance commitment subscale of the organizational commitment scale also reported by Meyer et al. [63]. The continuance commitment subscale consists of 6 items, which included “Right now, staying with my organization is a matter of necessity as much as desire”; “It would be very hard for me to leave my organization right now, even if I wanted to”; “Too much of my life would be disrupted if I decided I wanted to leave my organization now”; and “I feel that I have too few options to consider leaving my organization”. The reliability of the scale was α =  0.85.

#### Normative commitment.

Continuance commitment was measured using the continuance commitment subscale of the organizational commitment scale also reported by Meyer et al. [63]. The continuance commitment subscale consists of 6 items, which included “I do not feel any obligation to remain with my current employer” (r); “I would feel guilty if I left my organization now”; and “I would not leave my organization right now because I have a sense of obligation to the people in it.” The reliability of the scale was α =  0.91.

#### Perceived contract breach.

Perceived contract breach was measured using the 5-item scale reported by Robinson & Morrison [64]. Examples of items included in the scale are: “Almost all the promises made by my employer during recruitment have been kept so far (reversed)”; “I feel that my employer has come through in fulfilling the promises made to me when I was hired (reversed)”; “So far my employer has done an excellent job of fulfilling its promises to me (reversed)”; and “I have not received everything promised to me in exchange for my contributions”. The reliability of the scale was α =  0.95.

#### Feeling of violation.

Feeling of violation was measured using a 4-item scale also reported by Robinson & Morrison [64]. Examples of items included: “I feel a great deal of anger toward my organization”; “I feel betrayed by my organization”; “I feel that my organization has violated the contract between us”; and “I feel extremely frustrated by how I have been treated by my organization”. The reliability of the scale was α =  0.96.

#### Social desirability.

Finally, Social desirability was measured using the 4-item scale developed by Haghighat [114]. Examples of items included in the measure are: “Would you smile at people every time you meet them?”; “Do you always practice what you preach to people?”; “If you say that you will do something, do you always keep your promise, no matter how inconvenient it might be?”; and “Would you ever lie to people?”. The reliability of the scale was α =  0.68. Where applicable, items were reverse-coded for purposes of analysis. In Table 4 we present the sample descriptives.

### Scale testing

To assess the inclusion of one or more scale items, we present an abridged table of Loevinger coefficients, and the distribution of responses in Table 5. The second item of the Behavioral QQ scale, and the seventh item of the Emotional QQ scale exhibited lower values, which initially positioned them as potential candidates for deletion. However, we note that the coefficients for both these items were above 0.3. We further assessed the item loadings for the CFA in Table 6 and found the second Behavioral QQ item had a relatively lower loading, also positioning it as a potential candidate for deletion. As can be seen in Table 7, upon dropping item 2 from the Behavioral QQ scale, the cutoffs and loadings remained within an acceptable range. As can be seen in Table 8, when excluding Behavioral QQ item 3, the CFA improved slightly, and the reliabilities and *Hj_min* values also remained within the acceptable range. Based on our earlier discussion bearing on the process for determining scale fit, an SRMR/ *R*^*2*^ =  0.060/0.6467 =  0.092 indicates an adequate fit of the model to the data.

**Table 5:** Study 2-Scale tests Abridged item results list of Loevinger coefficient.

	1	2	3	4	5	Alpha - item	Loevinger Hj coefficient
QQ Behavioral 1	29.54%	34.21%	12.99%	15.13%	8.12%	0.83	0.67
***QQ*** *Behavioral 2*	*4.16%*	*6.70%*	*11.37%*	*35.94%*	*41.83%*	*0.90*	*0.34*
QQ Behavioral 3	21.22%	32.18%	12.59%	19.59%	14.42%	0.83	0.63
QQ Behavioral 4	19.49%	31.37%	20.61%	18.27%	10.25%	0.84	0.6
QQ Behavioral 5	22.23%	34.42%	17.36%	17.97%	8.02%	0.83	0.66
QQ Behavioral 6	14.42%	19.90%	23.55%	23.76%	18.38%	0.84	0.62
QQ Emotional 1	23.35%	34.92%	16.24%	15.33%	10.15%	0.86	0.49
QQ Emotional 2	19.39%	23.15%	26.40%	19.19%	11.88%	0.84	0.59
QQ Emotional 3	22.54%	28.53%	22.74%	18.38%	7.82%	0.87	0.41
QQ Emotional 4	19.19%	25.58%	15.03%	25.69%	14.52%	0.84	0.57
QQ Emotional 5	10.46%	16.65%	18.68%	31.17%	23.05%	0.84	0.58
QQ Emotional 6	12.39%	17.36%	20.71%	27.72%	21.83%	0.83	0.63
***QQ*** *Emotional 7*	*14.82%*	*27.01%*	*15.74%*	*26.09%*	*16.35%*	*0.88*	*0.37*

The scale items as candidates for dropping are bolded and italicized based on Loevinger cutoffs. The full table is available upon request.

**Table 6:** Study 2-Scale tests abridged item loadings.

Scale Item	Factor loading	s.e.	Intercept	s.e.	Error variance
QQ Behavioral 1	0.84	0.01	1.87	0.03	0.29
***QQ*** *Behavioral 2*	*0.33*	*0.02*	*3.74*	*0.11*	*0.89*
QQ Behavioral 3	0.80	0.01	2.00	0.04	0.36
QQ Behavioral 4	0.77	0.02	2.13	0.04	0.41
QQ Behavioral 5	0.85	0.01	2.06	0.04	0.27
QQ Emotional 1	0.77	0.01	2.37	0.05	0.40
QQ Emotional 2	0.65	0.02	1.99	0.04	0.58
QQ Emotional 3	0.81	0.01	2.20	0.04	0.35
QQ Emotional 4	0.55	0.02	2.11	0.04	0.69
QQ Emotional 5	0.78	0.01	2.14	0.04	0.39
QQ Emotional 6	0.78	0.01	2.64	0.06	0.39
QQ Emotional 7	0.86	0.01	2.50	0.05	0.27
OCBI 1	0.51	0.02	2.27	0.05	0.74
OCBI 2	0.82	0.01	3.68	0.10	0.33
OCBI 3	0.83	0.01	3.70	0.10	0.31
OCBI 4	0.65	0.02	2.61	0.06	0.58
OCBI 5	0.65	0.02	4.27	0.12	0.58
OCBI 6	0.77	0.02	3.82	0.10	0.40
OCBI 7	0.61	0.03	5.94	0.19	0.63
OCBO 1	0.43	0.03	3.51	0.08	0.81
OCBO 2	0.49	0.03	5.74	0.18	0.76
OCBO 3	0.52	0.02	3.41	0.08	0.73
OCBO 4	0.33	0.03	4.39	0.14	0.89
OCBO 5	0.43	0.03	3.88	0.09	0.82
OCBO 6	0.61	0.03	4.53	0.12	0.63
OCBO 7	0.50	0.03	4.62	0.13	0.75
Voice 1	0.88	0.01	3.45	0.09	0.22
Voice 2	0.86	0.01	3.14	0.08	0.26
Voice 3	0.76	0.02	3.51	0.10	0.42
Voice 4	0.77	0.02	4.09	0.12	0.41
Voice 5	0.78	0.02	3.40	0.09	0.40
Voice 6	0.88	0.01	3.37	0.09	0.22
Turnover Intention 1	0.95	0.01	1.78	0.03	0.11
Turnover Intention 2	0.87	0.01	1.83	0.04	0.24
Turnover Intention 3	0.61	0.02	1.86	0.04	0.62
Affective commitment 1	0.76	0.01	2.26	0.05	0.43
Affective commitment 2	0.74	0.02	1.92	0.04	0.45
Affective commitment 3	0.88	0.01	2.44	0.06	0.22
Affective commitment 4	0.88	0.01	2.18	0.05	0.23
Affective commitment 5	0.86	0.01	2.50	0.06	0.26
Affective commitment 6	0.88	0.01	2.31	0.05	0.23
Continuance commitment 1	0.61	0.02	2.50	0.06	0.63
Continuance commitment 2	0.64	0.02	2.37	0.05	0.59
Continuance commitment 3	0.58	0.02	2.62	0.06	0.67
Continuance commitment 4	0.89	0.01	2.05	0.04	0.21
Continuance commitment 5	0.47	0.02	2.04	0.04	0.78
Continuance commitment 6	0.87	0.01	2.15	0.04	0.24
Normative commitment 1	0.77	0.02	2.05	0.04	0.41
Normative commitment 2	0.75	0.02	2.06	0.04	0.43
Normative commitment 3	0.79	0.01	1.97	0.04	0.38
Normative commitment 4	0.85	0.01	2.14	0.05	0.28
Normative commitment 5	0.84	0.01	2.03	0.04	0.29
Normative commitment 6	0.80	0.01	2.14	0.04	0.36
Perceived contract breach 1	0.92	0.01	1.93	0.03	0.15
Perceived contract breach 2	0.94	0.01	1.91	0.03	0.12
Perceived contract breach 3	0.94	0.01	1.95	0.04	0.11
Perceived contract breach 4	0.79	0.02	1.80	0.03	0.37
Perceived contract breach 5	0.85	0.01	1.71	0.03	0.27
Feelings of violation	0.89	0.01	1.55	0.02	0.21
Feelings of violation	0.93	0.01	1.55	0.03	0.13
Feelings of violation	0.93	0.01	1.59	0.03	0.14
Feelings of violation	0.94	0.01	1.54	0.03	0.12
Social Desirability 1	0.42	0.03	3.37	0.09	0.82
Social Desirability 2	0.75	0.02	3.96	0.11	0.44
Social Desirability 3	0.75	0.03	4.33	0.12	0.44
Social Desirability 4	0.50	0.03	2.72	0.05	0.75

The scale items as candidates for dropping are bolded and italicized based on Loevinger cutoffs. The full table is available upon request.

**Table 7:** Study 2- Scale tests Abridged item of Loevinger coefficients with the second item from QQ behavioral scale dropped.

	Alpha - item	Loevinger Hj coefficient
QQ Behavioral 1	0.87	0.73
QQ Behavioral 3	0.88	0.68
QQ Behavioral 4	0.89	0.65
QQ Behavioral 5	0.87	0.71
QQ Behavioral 6	0.89	0.67
QQ Emotional 1	0.86	0.49
QQ Emotional 2	0.84	0.59
QQ Emotional 3	0.87	0.41
QQ Emotional 4	0.84	0.57
QQ Emotional 5	0.84	0.58
QQ Emotional 6	0.83	0.63
QQ Emotional 7	0.88	0.37

The scale items as candidates for dropping are bolded and italicized based on Loevinger cutoffs. The full table is available upon request.

**Table 8:** Study 2- Scale tests item reliabilities and CFA.

	n	alpha	H	Hj_min
QQ Behavioral	985	0.90	0.69	0.65
QQ Emotional	985	0.87	0.52	0.37
OCBI	985	0.86	0.57	0.51
OCBO	985	0.67	0.24	0.20
Voice	985	0.93	0.71	0.66
Turnover intention	985	0.84	0.67	0.56
Affective Commitment	985	0.93	0.72	0.65
Continuance Commitment	985	0.85	0.51	0.39
Normative Commitment	985	0.91	0.66	0.63
Perceived contract breach	985	0.95	0.82	0.76
Feelings of violation	985	0.96	0.88	0.85
Social desirability	985	0.68	0.38	0.28

Chi2 = 5789.56; df = 2013; chi2/df = 2.9; RMSEA = 0.044; SRMR = 0.060; CFI = 0.909.

Unbiased SRMR: *SRMR/ R*^*2*^ *= 0.060/0.6467 = 0.092 (adequately fitting)*.

The results from OLS analysis are presented in Table 9. Using OCBI, OCBO, and Turnover Intentions as outcomes, and using Voice; Affective Commitment; Continuance Commitment; Normative Commitment; Perceived contract breach; Feelings of the violation; and Social desirability as predictors, we tested the incremental validity, and direction of effects, of the MQQS measure. In Table 9 as expected for OCBI, in model 4 Behavioral QQ is negatively associated with OCBI (β =  -0.21, *p* <  0.01), in model 8 Behavioral QQ (β =  -0.16, *p* <  0.01) is negatively related to OCBO, and in model 12 Emotional QQ is positively related to turnover intentions (β =  0.09, *p* <  0.05). To facilitate the development of inferences bearing on the relevance of the MQQS subscales, the results from relative weights analysis are presented in Table 10.

**Table 9:** Study 2- OLS estimates.

	DV = OCBI				DV = OCBO				DV = Turnover Intention			
	(1)	(2)	(3)	(4)	(5)	(6)	(7)	(8)	(9)	(10)	(11)	(12)
QQ Behavioral		-0.21^***^		-0.21^***^		-0.16^***^		-0.16^***^		0.05^*^		-0.01
		(0.02)		(0.03)		(0.02)		(0.02)		(0.03)		(0.04)
QQ Emotional			-0.17^***^	-0.00			-0.13^***^	-0.00			0.08^***^	0.09^**^
			(0.02)	(0.03)			(0.02)	(0.03)			(0.03)	(0.04)
Voice	0.41^***^	0.34^***^	0.38^***^	0.34^***^	0.09^***^	0.04^**^	0.07^***^	0.04^**^	0.10^***^	0.12^***^	0.12^***^	0.11^***^
	(0.02)	(0.02)	(0.02)	(0.02)	(0.02)	(0.02)	(0.02)	(0.02)	(0.03)	(0.03)	(0.03)	(0.03)
Affective Commitment	0.07^**^	0.04	0.04	0.04	0.07^***^	0.04^*^	0.04^*^	0.04^*^	-0.55^***^	-0.55^***^	-0.54^***^	-0.54^***^
	(0.03)	(0.03)	(0.03)	(0.03)	(0.03)	(0.02)	(0.02)	(0.02)	(0.04)	(0.04)	(0.04)	(0.04)
Continuance Commitment	-0.02	-0.01	-0.00	-0.01	-0.03	-0.02	-0.01	-0.02	0.02	0.01	0.01	0.01
	(0.02)	(0.02)	(0.02)	(0.02)	(0.02)	(0.02)	(0.02)	(0.02)	(0.03)	(0.03)	(0.03)	(0.03)
Normative Commitment	0.10^***^	0.06^**^	0.08^***^	0.06^**^	-0.01	-0.05^**^	-0.03	-0.05^**^	-0.06^*^	-0.05	-0.05	-0.05
	(0.03)	(0.03)	(0.03)	(0.03)	(0.02)	(0.02)	(0.02)	(0.02)	(0.04)	(0.04)	(0.04)	(0.04)
Perceived contract breach	-0.03	-0.02	-0.01	-0.02	-0.02	-0.02	-0.01	-0.02	0.09^**^	0.08^**^	0.08^**^	0.08^**^
	(0.03)	(0.03)	(0.03)	(0.03)	(0.03)	(0.03)	(0.03)	(0.03)	(0.04)	(0.04)	(0.04)	(0.04)
Feelings of violation	0.05	0.05^*^	0.05^*^	0.05^*^	-0.03	-0.03	-0.03	-0.03	0.28^***^	0.28^***^	0.28^***^	0.28^***^
	(0.03)	(0.03)	(0.03)	(0.03)	(0.03)	(0.02)	(0.03)	(0.02)	(0.04)	(0.04)	(0.04)	(0.04)
Social desirability	0.13^***^	0.12^***^	0.12^***^	0.12^***^	0.21^***^	0.19^***^	0.19^***^	0.19^***^	-0.03	-0.03	-0.03	-0.03
	(0.03)	(0.03)	(0.03)	(0.03)	(0.02)	(0.02)	(0.02)	(0.02)	(0.04)	(0.04)	(0.04)	(0.04)
Constant	1.42^***^	2.44^***^	2.17^***^	2.44^***^	3.04^***^	3.83^***^	3.62^***^	3.83^***^	3.44^***^	3.21^***^	3.08^***^	3.10^***^
	(0.15)	(0.17)	(0.18)	(0.18)	(0.13)	(0.15)	(0.15)	(0.15)	(0.20)	(0.23)	(0.24)	(0.24)
Observations	985	985	985	985	985	985	985	985	985	985	985	985
Adjusted R2	0.44	0.49	0.47	0.49	0.21	0.28	0.25	0.28	0.62	0.62	0.62	0.62
R-square	0.44	0.50	0.47	0.50	0.22	0.29	0.25	0.29	0.62	0.62	0.63	0.63

Standard errors in parentheses; Unstandardized beta estimates are reported.

***p < 0.01,

**p < 0.05,

*  *p* < 0.1.

**Table 10:** Study 2- relative weight analysis.

	OCBI			OCBO			Turnover intention		
	Dominance Statistics	Std. Dominance Statistics (relative variance explained)	Ranking	Dominance Statistics	Std. Dominance Statistics (relative variance explained)	Ranking	Dominance Statistics	Std. Dominance Statistics (relative variance explained)	Ranking
Voice	0.19	0.38	1	0.03	0.10	4	0.02	0.02	7
Affective Commitment	0.05	0.09	4	0.02	0.08	5	0.21	0.34	1
Continuance Commitment	0.00	0.00	9	0.00	0.02	9	0.00	0.01	9
Normative Commitment	0.04	0.08	5	0.01	0.04	8	0.10	0.15	4
Perceived contract breach	0.01	0.03	7	0.01	0.05	6	0.11	0.17	3
Feelings of violation	0.01	0.02	8	0.01	0.05	7	0.12	0.19	2
Social desirability	0.04	0.07	6	0.07	0.26	2	0.01	0.01	8
QQ Behavioral	0.11	0.21	2	0.08	0.27	1	0.03	0.04	6
QQ Emotional	0.06	0.11	3	0.04	0.14	3	0.04	0.06	5
N	985			985			985		
Overall Fit Statistic	0.50			0.25			0.63		

As can be seen in Table 10, with respect to the interpretation of the potential redundancy of the MQQS scales, Behavioral and Emotional QQ rank 2^nd^ and 3^rd^ in predicting OCBI, the two scales rank 1^st^ and 3^rd^ in predicting OCBO, and rank 6^th^ and 5^th^ in predicting turnover intentions. The results from this relative-weights analysis provide evidence that the two dimensions of the MQQS are not completely redundant with one another, and that they also rank reasonably above several other scales in the MQQS nomological network with respect to the prediction of ‘going above and beyond’ behaviors such as organizational citizenship behavior, as well as employees’ turnover intentions.

In summary, the results reported in Study 2 provide support for the dimensionality of the MQQS Behavioral and QQ Emotional scales after dropping an item. Both CFA and relative weights analysis also lend support to the distinctiveness of the behavioral and emotional sub-scale. In Study 3, we report the results from tests of the MQQS using a broader, worldwide sample.

### Study 3. Cross-validation of the MQQS in diverse national/cultural settings

Ethics Commission approval for Study 3 was received from the institution of the 2^nd^ author on September 19, 2022 (and then an addendum to the original approval on October 10, 2022, where we updated the consent letter) with the approval number 22/2022 (and 23/2022 for the addendum). The participants provided their informed consent to participate in this study by clicking “Yes” on the consent form screen before proceeding to the survey. We informed them about the purpose of the study, the procedures, the time required, and their right to withdraw from the study. We assured them that all data collected would remain confidential and anonymous.

Building from the results from Study 2, on October 26, 2022, we conducted a global study where we posted the survey on TikTok, Facebook, and LinkedIn, and also distributed the survey through additional channels such as a newsletter from ISEG and Exame, which is a monthly national business magazine. To reach the sample size rationale intended, we also sent the survey out to more than 1.6 million general emails from companies headquartered in Europe and obtained from the Orbis dataset. The countries in the study are: Albania, Austria, Belarus, Belgium, Bosnia and Herzegovina, Bulgaria, Croatia, Cyprus, Czech Republic, Denmark, Estonia, Finland, France, Greece, Germany, Hungary, Iceland, Ireland, Italy, Latvia, Lithuania, Netherlands, Norway, Poland, Portugal, Romania, Serbia, Slovakia, Slovenia, Spain, Sweden, Switzerland, United Kingdom.

Based on the timeframes posted on the OSF pre-registration, we stopped the survey data collection on December 22, 2022. The data has limitations in terms of the anonymity of individuals responding to the survey, and also limited fidelity in terms of survey item response characteristics. In addition, when sending the invitation emails, we faced several challenges that are worthy of note: i) respondents receive many emails with requests for their attention, and are likely to have only a limited amount of time available to read and respond to these kinds of requests; ii) emails can be filtered directly to spam folders; iii) the origins of the email and the level of trust recipients are likely to have in the email are very likely to be questioned, and thus the email goes ignored; iv) the survey was written in English, which also may be a language that not all email recipients are comfortable with; and v) the survey was quite long which also increased the likelihood of it being left unfinished or discarded. Taken together, the dropout rate amongst recipients was quite high, and the total number of usable responses was lower than expected.

Of the 3,123 respondents to the emailed survey, we dropped responses from individuals reporting an age less than 18 (5 cases), and thereafter based on the pattern of responding on the first attention check item of “I work 14 months a year” which led to our dropping 812 cases. This initial cut was followed by a cut based on responses to the item “I have never used a computer”, which led to the elimination of an additional 25 cases, and “My current salary is paid in Kryptonite”, which led to the elimination of a further 40 observations. Finally, we dropped any cases with missing values, resulting in a final sample of 2,128 cases. The sample descriptives associated with participants’ responses to the scale items are presented in Table 11. We used MQQS scales identical to those reported in Study 2, with the exception that we excluded item 2 from the Behavioral QQ dimension of the MQQS.

**Table 11:** Study 3*-* descriptives.

		Mean	SD	Min	Max	1	2	3	4	5	6	7	8	9	10	11
1	QQ Behavioral	2.12	1.11	1.00	5.00	1.00										
2	QQ Behavioral	2.48	0.97	1.00	5.00	0.74^*^	1.00									
3	OCBI	4.06	0.79	1.00	5.00	-0.44^*^	-0.36^*^	1.00								
4	OCBO	4.07	0.66	1.00	5.00	-0.40^*^	-0.36^*^	0.49^*^	1.00							
5	Voice	2.52	1.24	1.00	5.00	0.49^*^	0.48^*^	-0.28^*^	-0.29^*^	1.00						
6	Turnover intention	4.10	0.86	1.00	5.00	-0.47^*^	-0.39^*^	0.54^*^	0.43^*^	-0.34^*^	1.00					
7	Affective Commitment	3.45	1.13	1.00	5.00	-0.57^*^	-0.55^*^	0.40^*^	0.32^*^	-0.69^*^	0.47^*^	1.00				
8	Continuance Commitment	2.97	0.96	1.00	5.00	0.18^*^	0.20^*^	-0.06^*^	-0.08^*^	0.20^*^	-0.04^*^	-0.07^**^	1.00			
9	Normative Commitment	3.09	1.11	1.00	5.00	-0.46^*^	-0.45^*^	0.35^*^	0.23^*^	-0.56^*^	0.32^*^	0.68^*^	0.03	1.00		
10	Perceived contract breach	2.35	1.12	1.00	5.00	0.38^*^	0.41^*^	-0.26^*^	-0.25^*^	0.62^*^	-0.27^*^	-0.58^*^	0.17^*^	-0.52^*^	1.00	
11	Feelings of violation	1.73	1.08	1.00	5.00	0.40^*^	0.40^*^	-0.24^*^	-0.25^*^	0.62^*^	-0.26^*^	-0.52^*^	0.20^*^	-0.46^*^	0.69^*^	1.00
12	Social desirability	3.84	0.66	1.00	5.00	-0.23^*^	-0.21^*^	0.33^*^	0.40^*^	-0.17^*^	0.32^*^	0.22^*^	-0.04	0.15^*^	-0.15^*^	-0.13^*^

*N*=2,128;

*p<0.05.

As can be seen in Table 12, the *Hj min* values were all above the recommended cutoffs, and all of the scale reliabilities also were above acceptable cutoff levels. The CFA Chi2 =  11350.65; df =  2013; chi2/df =  5.6; RMSEA =  0.047; SRMR =  0.066; CFI =  0.875, with SRMR/*R*^*2*^ =  0.066/0.607 = 0.108 (close to adequately fitting). In Table 13 we list the factor loadings.

**Table 12:** Study 3- Scale tests Study 2- Item reliabilities and CFA.

	n	alpha	H	*Hj_min*
QQ Behavioral	2,128	0.90	0.67	0.63
QQ Emotional	2,128	0.85	0.47	0.31
OCBI	2,128	0.88	0.55	0.48
OCBO	2,128	0.71	0.27	0.24
Voice	2,128	0.92	0.68	0.65
Turnover intention	2,128	0.80	0.61	0.49
Affective Commitment	2,128	0.90	0.61	0.55
Continuance Commitment	2,128	0.78	0.39	0.33
Normative Commitment	2,128	0.87	0.55	0.49
Perceived contract breach	2,128	0.93	0.75	0.66
Feelings of violation	2,128	0.94	0.84	0.82
Social desirability	2,128	0.59	0.28	0.19

Chi2 = 11350.65; df = 2013; chi2/df = 5.6; RMSEA = 0.047; SRMR = 0.066; CFI = 0.875.

Unbiased SRMR: SRMR/ *R*^*2*^ = 0.066/0.607= 0.108 (close to adequately fitting).

**Table 13:** Study 3- Scale tests - item loadings.

Scale Item	Factor loading	s.e.	Intercept	s.e.	Error variance
QQ Behavioral 1	0.84	0.01	1.56	0.02	0.29
QQ Behavioral 2	0.79	0.01	1.52	0.02	0.38
QQ Behavioral 3	0.79	0.01	1.65	0.02	0.37
QQ Behavioral 4	0.81	0.01	1.60	0.02	0.34
QQ Behavioral 5	0.76	0.01	1.71	0.02	0.42
QQ Emotional 1	0.52	0.02	1.74	0.02	0.73
QQ Emotional 2	0.71	0.01	1.78	0.02	0.50
QQ Emotional 3	0.60	0.01	1.91	0.03	0.64
QQ Emotional 4	0.78	0.01	1.78	0.02	0.40
QQ Emotional 5	0.82	0.01	1.98	0.03	0.33
QQ Emotional 6	0.85	0.01	1.83	0.02	0.28
QQ Emotional 7	0.42	0.02	1.92	0.02	0.82
OCBI 1	0.75	0.01	4.20	0.09	0.43
OCBI 2	0.80	0.01	4.09	0.08	0.37
OCBI 3	0.63	0.01	2.83	0.05	0.60
OCBI 4	0.70	0.01	4.37	0.09	0.51
OCBI 5	0.76	0.01	3.99	0.08	0.42
OCBI 6	0.70	0.01	3.34	0.06	0.51
OCBI 7	0.66	0.02	4.95	0.11	0.56
OCBO 1	0.53	0.02	3.40	0.06	0.72
OCBO 2	0.56	0.02	4.62	0.11	0.69
OCBO 3	0.51	0.02	3.08	0.05	0.74
OCBO 4	0.46	0.02	3.99	0.08	0.79
OCBO 5	0.43	0.02	3.41	0.06	0.82
OCBO 6	0.52	0.02	3.86	0.08	0.72
OCBO 7	0.51	0.02	3.81	0.07	0.74
Voice 1	0.80	0.01	4.18	0.09	0.36
Voice 2	0.84	0.01	3.87	0.08	0.30
Voice 3	0.77	0.01	4.09	0.09	0.41
Voice 4	0.81	0.01	4.17	0.09	0.34
Voice 5	0.83	0.01	3.93	0.08	0.32
Voice 6	0.84	0.01	3.94	0.09	0.30
Turnover Intention 1	0.91	0.01	1.83	0.02	0.18
Turnover Intention 2	0.86	0.01	1.68	0.02	0.26
Turnover Intention 3	0.57	0.02	1.70	0.02	0.68
Affective commitment 1	0.66	0.01	2.29	0.03	0.56
Affective commitment 2	0.63	0.01	2.15	0.03	0.60
Affective commitment 3	0.87	0.01	2.66	0.04	0.25
Affective commitment 4	0.88	0.01	2.65	0.04	0.22
Affective commitment 5	0.86	0.01	2.65	0.04	0.26
Affective commitment 6	0.70	0.01	2.42	0.04	0.50
Continuance commitment 1	0.49	0.02	2.43	0.04	0.76
Continuance commitment 2	0.58	0.02	2.29	0.04	0.67
Continuance commitment 3	0.62	0.01	2.31	0.04	0.61
Continuance commitment 4	0.78	0.01	2.00	0.03	0.39
Continuance commitment 5	0.50	0.02	1.88	0.02	0.75
Continuance commitment 6	0.68	0.01	1.98	0.03	0.54
Normative commitment 1	0.66	0.01	2.11	0.03	0.57
Normative commitment 2	0.63	0.01	2.13	0.03	0.60
Normative commitment 3	0.67	0.01	1.94	0.03	0.54
Normative commitment 4	0.81	0.01	2.31	0.03	0.34
Normative commitment 5	0.79	0.01	2.34	0.04	0.37
Normative commitment 6	0.76	0.01	2.17	0.03	0.42
Perceived contract breach 1	0.91	0.01	1.93	0.02	0.17
Perceived contract breach 2	0.94	0.01	1.93	0.02	0.11
Perceived contract breach 3	0.93	0.00	1.97	0.02	0.13
Perceived contract breach 4	0.69	0.01	1.83	0.02	0.53
Perceived contract breach 5	0.76	0.01	1.60	0.02	0.42
Feelings of violation	0.90	0.01	1.52	0.02	0.19
Feelings of violation	0.95	0.01	1.47	0.02	0.11
Feelings of violation	0.86	0.01	1.51	0.02	0.26
Feelings of violation	0.89	0.01	1.43	0.02	0.20
Social Desirability 1	0.46	0.02	4.04	0.08	0.79
Social Desirability 2	0.70	0.02	4.24	0.08	0.51
Social Desirability 3	0.66	0.02	4.67	0.09	0.57
Social Desirability 4	0.30	0.03	2.84	0.04	0.91

Next, we proceeded with the OLS analysis. As can be seen in Table 14, for OCBI, in model 4 Behavioral QQ was negatively associated with OCBI (β =  -0.11, *p* <  0.01), in model 8 Behavioral QQ (β =  -0.09, *p* <  0.01) and Emotional QQ (β =  -0.06, *p* <  0.01) both were negatively related OCBO, and in model 12 Behavioral QQ was not associated with turnover intentions (β =  0.04, *p* >  0.10).

**Table 14:** Study 3- OLS estimates.

	DV = OCBI				DV = OCBO				DV = Turnover Intention			
	(1)	(2)	(3)	(4)	(5)	(6)	(7)	(8)	(9)	(10)	(11)	(12)
QQ Behavioral		-0.12^***^		-0.11^***^		-0.12^***^		-0.09^***^		0.07^***^		0.04
		(0.02)		(0.02)		(0.01)		(0.02)		(0.02)		(0.02)
QQ Emotional			-0.08^***^	-0.01			-0.11^***^	-0.06^***^			0.07^***^	0.05^*^
			(0.02)	(0.02)			(0.02)	(0.02)			(0.02)	(0.03)
Voice	0.38^***^	0.34^***^	0.36^***^	0.34^***^	0.21^***^	0.18^***^	0.19^***^	0.18^***^	-0.03	-0.00	-0.01	-0.00
	(0.02)	(0.02)	(0.02)	(0.02)	(0.02)	(0.02)	(0.02)	(0.02)	(0.02)	(0.02)	(0.02)	(0.02)
Affective Commitment	0.06^***^	0.02	0.04^*^	0.02	0.04^**^	0.00	0.01	-0.01	-0.42^***^	-0.40^***^	-0.40^***^	-0.40^***^
	(0.02)	(0.02)	(0.02)	(0.02)	(0.02)	(0.02)	(0.02)	(0.02)	(0.02)	(0.02)	(0.02)	(0.02)
Continuance Commitment	-0.03^*^	-0.01	-0.02	-0.01	-0.02	-0.00	-0.00	0.00	0.13^***^	0.12^***^	0.12^***^	0.11^***^
	(0.02)	(0.02)	(0.02)	(0.02)	(0.01)	(0.01)	(0.01)	(0.01)	(0.02)	(0.02)	(0.02)	(0.02)
Normative Commitment	0.10^***^	0.09^***^	0.09^***^	0.08^***^	0.00	-0.02	-0.01	-0.02	-0.12^***^	-0.11^***^	-0.11^***^	-0.11^***^
	(0.02)	(0.02)	(0.02)	(0.02)	(0.02)	(0.02)	(0.02)	(0.02)	(0.02)	(0.02)	(0.02)	(0.02)
Perceived contract breach	-0.00	-0.01	0.00	-0.01	-0.02	-0.02	-0.01	-0.02	0.17^***^	0.17^***^	0.17^***^	0.17^***^
	(0.02)	(0.02)	(0.02)	(0.02)	(0.02)	(0.02)	(0.02)	(0.02)	(0.02)	(0.02)	(0.02)	(0.02)
Feelings of violation	0.00	0.02	0.01	0.02	-0.05^***^	-0.04^**^	-0.04^***^	-0.04^**^	0.27^***^	0.26^***^	0.27^***^	0.26^***^
	(0.02)	(0.02)	(0.02)	(0.02)	(0.02)	(0.02)	(0.02)	(0.02)	(0.02)	(0.02)	(0.02)	(0.02)
Social desirability	0.19^***^	0.18^***^	0.18^***^	0.18^***^	0.28^***^	0.27^***^	0.27^***^	0.27^***^	-0.00	0.00	0.00	0.00
	(0.02)	(0.02)	(0.02)	(0.02)	(0.02)	(0.02)	(0.02)	(0.02)	(0.03)	(0.03)	(0.03)	(0.03)
Constant	1.39^***^	1.92^***^	1.69^***^	1.93^***^	2.19^***^	2.75^***^	2.66^***^	2.85^***^	3.23^***^	2.93^***^	2.93^***^	2.85^***^
	(0.12)	(0.14)	(0.14)	(0.15)	(0.11)	(0.13)	(0.12)	(0.13)	(0.15)	(0.18)	(0.18)	(0.18)
Observations	2,128	2,128	2,128	2,128	2,128	2,128	2,128	2,128	2,128	2,128	2,128	2,128
Adjusted R2	0.36	0.37	0.36	0.37	0.28	0.30	0.30	0.31	0.59	0.59	0.59	0.60
R-square	0.36	0.37	0.36	0.37	0.28	0.31	0.30	0.31	0.59	0.60	0.60	0.60

Standard errors in parentheses.

***p < 0.01,

**p < 0.05,

*  *p* < 0.1.

To interpret the incremental validity of the dimensions of the MQQS, as can be seen in Table 15, Behavioral QQ ranked 2nd among the 9 scales included in the analysis in explaining variation in OCBI, and Emotional QQ ranked 6^th^ in explaining variation in OCBI, and 3^rd^ and 4^th^ respectively for relative variance explained OCBO. For turnover intentions, Behavioral and Emotional QQ ranked 5^th^ and 6^th^ respectively, explaining a higher amount of relative variance than voice behavior, continuance commitment, or social desirability. In summary, Study 3 confirmed findings from Study 2.

**Table 15:** Study 3- relative weight analysis.

	OCBI			OCBO			Turnover intention		
	Dominance Statistics	Std. Dominance Statistics (relative variance explained)	Ranking	Dominance Statistics	Std. Dominance Statistics (relative variance explained)	Ranking	Dominance Statistics	Std. Dominance Statistics (relative variance explained)	Ranking
Voice	0.15	0.41	1	0.08	0.26	2	0.02	0.04	7
Affective Commitment	0.04	0.10	4	0.02	0.07	5	0.16	0.27	1
Continuance Commitment	0.00	0.00	9	0.00	0.00	9	0.01	0.02	8
Normative Commitment	0.03	0.08	5	0.01	0.03	8	0.08	0.13	4
Perceived contract breach	0.01	0.03	7	0.01	0.04	7	0.11	0.18	3
Feelings of violation	0.01	0.02	8	0.01	0.04	6	0.12	0.20	2
Social desirability	0.05	0.12	3	0.09	0.30	1	0.00	0.01	9
0QQ Behavioral	0.06	0.015	2	0.05	0.16	3	0.05	0.08	5
QQ Emotional	0.03	0.08	6	0.03	0.11	4	0.05	0.08	6
N	2,128			2,128			2,128		
Overall Fit Statistic	0.37			0.31			0.60		

### Study 4. Cross-validation of the MQQS in a field setting

The Ethics Commission approval for Study 4 was received from was received from the institution of the 2^nd^ author on September 19, 2022 (and then again in an addendum on October 10, 2022, where we updated the consent letter) with an approval number 22/2022 (and 23/2022 for the addendum). The participants provided their informed consent to participate in this study by clicking “Yes” on the consent form screen before proceeding to the survey. We informed them about the purpose of the study, the procedures, the time required, and their right to withdraw from the study. We assured them that all data collected would remain confidential and anonymous.

In Studies 2 and 3, we did not control for a wide range of demographic or firm-related characteristics with the potential to play a role in the relationships between MQQS and the outcome variables in focus in the study model we tested. Therefore, to provide a more conservative test of the operation of the MQQS, in Study 4 we drew on a sample of Portuguese employees. In Portugal, public-limited companies and limited liability companies, irrespective of their size or age are required to disclose their annual financial information using a form called IES (*Informação Empresarial Simplificada*), which is a composite simplification of company information for the past year. By law, these statements are officially certified by a public accountant and are recognized as being a highly reliable depiction of the company’s profile.

These archival financial data are available through Informa D&B. In addition, we also obtained the public general email for the largest 30,000 public-limited companies and limited liability companies in Portugal. Using this database, we sent an email invitation to potential participants in this study, on January 3, 2023. In the service of enhancing rates of participation, we promised a donation of 0.5 Euros to a charity chosen by the respondent, pursuant to the completion of the study survey. We obtained a total of 2,366 complete responses (i.e., representing a participation rate of approximately 8%). From the 2,366 responses we obtained, 57 responded “yes” to the attention check item on “My salary is paid in Kryptonite” and were therefore dropped from the sample. We also dropped responses from participants who indicated that they were not employed full-time, self-identified as self-employed, or who returned surveys with any missing values for the financial data. Following this conservative culling approach, based on case wise individual-level deletion, and firm level control variables, our final sample included a total of 1,897 full-time employees.

As a preliminary confirmation of scale validity, we conducted the scale reliability test for all constructs in the model. As can be seen from the results presented in Table 16(a), the results from this analysis indicated that the *Hj_min* was low for item 7 of the Emotional QQ dimension of the MQQS. As can be seen from the results presented in Table 16(b), the *Hj_min* values remained above cutoff levels following removal of item 7 of the Emotional QQ dimension. The results from CFA, based on evidence reflecting on the interpretation of SRMR and RMSEA (discussed earlier in the analytics decision section), was acceptable: Chi2 =  10638.64; df =  1949; chi2/df =  5.5; RMSEA =  0.048; SRMR =  0.078; CFI =  0.849, with unbiased SRMR: SRMR/ *R*^*2*^ =  0.078/0.582 = 0.134 (close to adequately fitting). The factor loadings of the revised scale based on the CFA are presented in Table 17.

**Table 16:** Study 4- scale tests.

	n	alpha	H	Hj_min
(a) Study 4- item reliabilities and CFA.				
QQ Behavioral	1,897	0.84	0.54	0.52
***QQ*** *Emotional (7 items)*	*1,897*	*0.80*	*0.40*	*0.16 (item 7)*
OCBI	1,897	0.87	0.54	0.47
OCBO	1,897	0.68	0.25	0.22
Voice	1,897	0.90	0.64	0.59
Turnover intention	1,897	0.82	0.63	0.53
Affective Commitment	1,897	0.86	0.54	0.48
Continuance Commitment	1,897	0.70	0.30	0.22
Normative Commitment	1,897	0.84	0.50	0.33
Perceived contract breach	1,897	0.91	0.70	0.54
Feelings of violation	1,897	0.94	0.82	0.80
Social desirability	1,897	0.65	0.33	0.22
CFA: Chi2 = 10938.22; df = 2013; chi2/df = 5.4; RMSEA = 0.048; SRMR = 0.078; CFI = 0.846				
Unbiased SRMR: SRMR/* R*^2^ = 0.078/0.582= 0.134 (close to adequately fitting).				
(b) Study 4- scale test with 7th removed from QQ emotional items.				
QQ Behavioral	1,897	0.84	0.54	0.52
QQ Emotional (*6 items*)	1,897	0.83	0.49	0.33
OCBI	1,897	0.87	0.54	0.47
OCBO	1,897	0.68	0.25	0.22
Voice	1,897	0.90	0.64	0.59
Turnover intention	1,897	0.82	0.63	0.53
Affective Commitment	1,897	0.86	0.54	0.48
Continuance Commitment	1,897	0.70	0.30	0.22
Normative Commitment	1,897	0.84	0.50	0.33
Perceived contract breach	1,897	0.91	0.70	0.54
Feelings of violation	1,897	0.94	0.82	0.80
Social desirability	1,897	0.65	0.33	0.22

CFA: Chi2 = 10638.64; df = 1949; chi2/df = 5.5; RMSEA = 0.048; SRMR = 0.078; CFI = 0.849

Unbiased SRMR: SRMR/ *R*^*2*^ = 0.078/0.582= 0.134 (close to adequately fitting).

**Table 17:** Study 4- item loadings.

Scale Item	Factor loading	s.e.	Intercept	s.e.	Error variance
QQ Behavioral 1	0.74	0.02	1.57	0.02	0.45
QQ Behavioral 2	0.68	0.02	1.43	0.02	0.54
QQ Behavioral 3	0.69	0.02	1.50	0.02	0.53
QQ Behavioral 4	0.77	0.01	1.58	0.02	0.40
QQ Behavioral 5	0.71	0.02	1.59	0.02	0.49
QQ Emotional 1	0.45	0.02	1.64	0.02	0.80
QQ Emotional 2	0.68	0.01	1.60	0.02	0.54
QQ Emotional 3	0.62	0.01	1.79	0.03	0.61
QQ Emotional 4	0.76	0.01	1.69	0.02	0.42
QQ Emotional 5	0.75	0.01	1.78	0.02	0.43
QQ Emotional 6	0.81	0.01	1.69	0.02	0.35
OCBI 1	0.74	0.02	5.50	0.15	0.45
OCBI 2	0.72	0.02	4.76	0.11	0.48
OCBI 3	0.60	0.02	3.65	0.07	0.64
OCBI 4	0.68	0.01	4.01	0.08	0.54
OCBI 5	0.81	0.01	5.55	0.16	0.34
OCBI 6	0.69	0.02	3.93	0.08	0.53
OCBI 7	0.71	0.02	5.42	0.17	0.50
OCBO 1	0.48	0.02	4.09	0.09	0.77
OCBO 2	0.56	0.03	6.11	0.21	0.69
OCBO 3	0.41	0.03	4.02	0.09	0.83
OCBO 4	0.50	0.03	5.76	0.17	0.75
OCBO 5	0.48	0.03	4.73	0.11	0.77
OCBO 6	0.49	0.02	4.18	0.09	0.76
OCBO 7	0.46	0.02	4.11	0.08	0.79
Voice 1	0.76	0.01	4.52	0.10	0.42
Voice 2	0.79	0.01	4.06	0.08	0.38
Voice 3	0.78	0.01	4.88	0.11	0.40
Voice 4	0.82	0.01	5.12	0.11	0.33
Voice 5	0.70	0.02	4.10	0.10	0.51
Voice 6	0.83	0.01	4.67	0.10	0.30
Turnover Intention 1	0.90	0.01	1.79	0.03	0.19
Turnover Intention 2	0.84	0.01	1.58	0.02	0.30
Turnover Intention 3	0.62	0.02	1.81	0.03	0.61
Affective commitment 1	0.62	0.01	2.51	0.04	0.61
Affective commitment 2	0.59	0.01	3.18	0.05	0.65
Affective commitment 3	0.71	0.02	2.94	0.06	0.50
Affective commitment 4	0.86	0.01	3.51	0.07	0.27
Affective commitment 5	0.85	0.01	3.36	0.07	0.28
Affective commitment 6	0.68	0.01	2.88	0.05	0.53
Continuance commitment 1	0.41	0.02	2.57	0.04	0.83
Continuance commitment 2	0.76	0.02	2.76	0.05	0.42
Continuance commitment 3	0.76	0.02	2.51	0.04	0.43
Continuance commitment 4	0.45	0.02	2.16	0.03	0.80
Continuance commitment 5	0.33	0.02	2.01	0.03	0.89
Continuance commitment 6	0.23	0.02	1.99	0.03	0.94
Normative commitment 1	0.44	0.02	2.48	0.04	0.81
Normative commitment 2	0.72	0.01	2.28	0.04	0.48
Normative commitment 3	0.72	0.01	2.09	0.03	0.49
Normative commitment 4	0.72	0.01	3.19	0.06	0.48
Normative commitment 5	0.83	0.01	2.75	0.05	0.32
Normative commitment 6	0.74	0.01	2.72	0.04	0.45
Perceived contract breach 1	0.95	0.01	1.86	0.03	0.09
Perceived contract breach 2	0.97	0	1.84	0.03	0.06
Perceived contract breach 3	0.95	0	1.86	0.03	0.10
Perceived contract breach 4	0.51	0.02	1.71	0.03	0.74
Perceived contract breach 5	0.68	0.01	1.63	0.02	0.54
Feelings of violation	0.86	0.01	1.58	0.02	0.27
Feelings of violation	0.92	0.01	1.50	0.02	0.15
Feelings of violation	0.89	0.01	1.55	0.02	0.21
Feelings of violation	0.89	0.01	1.43	0.02	0.21
Social Desirability 1	0.55	0.02	4.16	0.08	0.69
Social Desirability 2	0.77	0.02	4.70	0.11	0.41
Social Desirability 3	0.67	0.02	4.99	0.11	0.56
Social Desirability 4	0.32	0.03	3.86	0.08	0.90

Descriptive statistics associated with the MQQS are presented in Table 17. It is important to note here that the firm-level controls were drawn from archival financial data and were not self-reported. With respect to individual level difference factors with potential to drive variation in the propensity to engage in Behavioral and Emotional Quiet Quitting, we controlled for participant age (1: 18-24; 2: 25-39; 3: 40-64; and 5: 65+) and sex (1-female; 2-male) [115]. Because marital status has the potential to influence career preferences and decisions relating to employment [116], we controlled for marital status (i.e., single, married or in a union, divorced, or widowed). Educational level also has the potential to influence perceptions of alternative employment options [117], and thus quiet quitting dynamics as well. We therefore also controlled for education level (i.e., 12^th^ grade or below; bachelors; masters; post-graduate; or doctorate). Because career level and compensation also may play a role in decisions about Quiet Quitting [118], we included income as a categorical variable (i.e., 1- less than 20,000 Euros to 7-150,000 Euros or more), as well as participants’ hierarchical position within their firm (i.e., Administration; Board of Directors, Management and other Corporate Bodies; Middle management; First line management; or No management position). Finally, because the length of time in the current role can play a role in Quiet Quitting, we also controlled for tenure (in years) in the current firm. As industries may systematically vary in terms of Quiet Quitting dynamics, we clustered standard errors by two-digit industry codes. Descriptive statistics are presented in Tables 18–20.

**Table 18:** Study 4 –descriptives.

	Mean	SD	Min	p25	Median	p75	Max
QQ Behavioral	1.66	0.85	1.00	1.00	1.40	2.00	5.00
QQ Behavioral	2.11	0.92	1.00	1.33	2.00	2.67	5.00
OCBI	4.26	0.70	1.00	3.86	4.43	4.86	5.00
OCBO	4.37	0.55	1.00	4.14	4.43	4.71	5.00
Voice	4.22	0.76	1.00	3.83	4.33	5.00	5.00
Turnover intention	2.37	1.18	1.00	1.33	2.33	3.00	5.00
Affective Commitment	3.87	0.97	1.00	3.17	4.00	4.67	5.00
Continuance Commitment	3.13	0.85	1.00	2.50	3.17	3.67	5.00
Normative Commitment	3.42	1.00	1.00	2.83	3.50	4.17	5.00
Perceived contract breach	2.24	1.08	1.00	1.00	2.00	3.00	5.00
Feelings of violation	1.51	0.92	1.00	1.00	1.00	1.75	5.00
Social desirability	4.07	0.64	1.00	3.75	4.00	4.50	5.00
Sex (1-female; 2-male)	1.39	0.49	1.00	1.00	1.00	2.00	2.00
Age (1: 18-24; 2: 25-39; 3: 40-64; and 5: 65^+^)	3.68	0.56	2.00	3.00	4.00	4.00	5.00
Married or in a union (ref. single)	0.70	0.46	0.00	0.00	1.00	1.00	1.00
Divorced	0.10	0.30	0.00	0.00	0.00	0.00	1.00
Widowed	0.01	0.08	0.00	0.00	0.00	0.00	1.00
Bachelors (ref. 12th grade or below)	0.43	0.50	0.00	0.00	0.00	1.00	1.00
Masters	0.15	0.36	0.00	0.00	0.00	0.00	1.00
Post-graduate degree	0.13	0.34	0.00	0.00	0.00	0.00	1.00
Doctorate	0.01	0.11	0.00	0.00	0.00	0.00	1.00
Income (1- less than 20,000 Euros t- 7-150,000 Euros or more	1.88	1.23	1.00	1.00	2.00	2.00	7.00
Middle management (ref. Administration; Board of Directors, Management and other Corporate Bodies)	0.31	0.46	0.00	0.00	0.00	1.00	1.00
First line management	0.18	0.38	0.00	0.00	0.00	0.00	1.00
No management position	0.27	0.44	0.00	0.00	0.00	1.00	1.00
Tenure in the firm	12.32	9.20	0.00	5.00	10.00	19.00	56.00
Log of sales	8.91	6.38	0.00	0.00	12.19	14.02	19.90
Log of employees	2.66	1.19	0.00	1.90	2.49	3.27	9.24
Net income (in ‘00,000 Euros)	2.91	23.17	-91.39	0.03	0.29	1.09	634.99
Log of staff expenses	12.28	2.12	0.00	11.59	12.32	13.24	18.89

N =  1,897

**Table 19:** Study 4 –descriptives.

	Variables	(1)	(2)	(3)	(4)	(5)	(6)	(7)	(8)	(9)	(10)	(11)	(12)	(13)	(14)	(15)	(16)	(17)
1	QQ Behavioral	1.00																
2	QQ Emotional	0.67*	1.00															
3	OCBI	-0.39*	-0.31*	1.00														
4	OCBO	-0.30*	-0.27*	0.53*	1.00													
5	Voice	-0.38*	-0.28*	0.54*	0.47*	1.00												
6	Turnover intention	0.41*	0.44*	-0.25*	-0.22*	-0.27*	1.00											
7	Affective Commitment	-0.50*	-0.43*	0.37*	0.30*	0.43*	-0.66*	1.00										
8	Continuance Commitment	0.08*	0.17*	0.05*	0.03	0.01	0.06*	0.04	1.00									
9	Normative Commitment	-0.37*	-0.32*	0.31*	0.18*	0.26*	-0.54*	0.62*	0.22*	1.00								
10	Perceived contract breach	0.34*	0.36*	-0.25*	-0.18*	-0.24*	0.61*	-0.52*	0.04	-0.53*	1.00							
11	Feelings of violation	0.43*	0.42*	-0.22*	-0.23*	-0.25*	0.61*	-0.51*	0.10*	-0.46*	0.62*	1.00						
12	Social desirability	-0.28*	-0.29	0.41*	0.40*	0.38*	-0.22*	0.26*	0.02	0.21*	-0.21*	-0.20*	1.00					
13	Sex (1-female; 2-male)	0.06*	-0.03	-0.06*	-0.06*	0.04	-0.07*	0.08*	-0.07*	0.07*	-0.08*	-0.07*	-0.01	1.00				
14	Age (1: 18-24; 2: 25-39; 3: 40-64; and 5: 65^+^)	-0.07*	-0.16*	0.07*	0.07*	0.13*	-0.10*	0.17*	0.05*	0.09*	-0.02	-0.03	0.08*	0.16*	1.00			
15	Married or in a union (ref. single)	-0.09*	-0.11*	0.07*	0.04	0.07*	-0.08*	0.13*	0.00	0.09*	-0.08*	-0.06*	0.07*	0.12*	0.27*	1.00		
16	Divorced	0.01	-0.01	-0.01	0.02	0.01	0.00	0.00	0.02	0.00	0.04	0.04	0.02	-0.03	0.18*	-0.50	1.00	
17	Widowed	0.01	-0.01	-0.01	-0.01	0.02	-0.02	0.01	-0.02	0.01	0.00	0.02	-0.02	0.00	0.09*	-0.12	-0.03	1.00
18	Bachelors (ref. 12th grade or below)	0.02	0.02	-0.06*	-0.04	-0.05*	0.02	-0.05*	0.03	0.00	0.04	0.03	-0.01	-0.01	-0.03	-0.02	0.00	-0.02
19	Masters	0.00	0.05*	0.03	0.02	0.08*	0.00	0.02	-0.09*	-0.02	0.00	-0.02	0.02	0.02	-0.15*	-0.08	-0.03	-0.01
20	Post-graduate degree	-0.04	-0.03	0.02	0.04	0.07*	0.03	0.02	-0.04	-0.03	0.00	0.00	0.01	-0.01	0.05*	0.05	-0.01	-0.03
21	Doctorate	0.01	-0.04	0.02	0.02	0.05*	-0.05*	0.04	-0.01	0.01	-0.02	-0.01	0.02	0.09*	0.10*	0.03	0.01	0.06
22	Income (1- less than 20,000 Euros t- 7-150,000 Euros or more	-0.10*	-0.15*	0.07*	0.05*	0.20*	-0.17*	0.21*	-0.06*	0.12*	-0.13*	-0.10*	0.07*	0.29*	0.28*	0.15	0.03	0.02
23	Middle management (ref. Administration; Board of Directors, Management and other Corporate Bodies)	-0.13*	-0.06*	0.03	0.04	0.10*	-0.03	0.11*	-0.03	0.05*	-0.02	-0.08*	0.01	-0.01	0.01	0.05	-0.03	-0.02
24	First line management	0.09*	0.09*	-0.03	-0.02	-0.04	0.11*	-0.12*	0.00	-0.11*	0.08*	0.07*	-0.06*	-0.06*	-0.10*	-0.05	-0.01	-0.02
25	No management position	0.20*	0.17*	-0.12*	-0.08*	-0.26*	0.20*	-0.32*	0.02	-0.25*	0.19*	0.17*	-0.06*	-0.26*	-0.18*	-0.12	-0.03	0.03
26	Tenure in the firm	-0.05*	-0.14*	0.05*	0.02	0.03	-0.10*	0.14*	0.10*	0.12*	-0.02	0.00	0.03	0.12*	0.51*	0.17	0.11	0.06
27	Log of sales	-0.05*	-0.02	0.02	0.00	-0.02	-0.01	0.02	0.04	0.02	-0.01	0.00	-0.02	-0.02	-0.05*	0.00	-0.02	-0.03
28	Log of employees	0.02	0.04	-0.02	0.01	0.03	0.08*	-0.05*	0.00	-0.06*	0.10*	0.07*	-0.05*	-0.11*	-0.04	-0.01	-0.05	0.03
29	Net income (in ‘00,000 Euros)	-0.02	0.02	0.02	0.04	-0.01	0.01	-0.02	-0.02	-0.05*	0.03	0.03	0.01	0.01	0.04	0.03	-0.01	0.01
30	Log of staff expenses	0.01	0.02	0.01	0.01	0.04	0.03	-0.02	-0.02	-0.02	0.05*	0.05*	-0.04	-0.06*	-0.01	-0.01	-0.01	0.02

**Table 20:** Study 4 –descriptives.

	Variables	(18)	(19)	(20)	(21)	(22)	(23)	(24)	(25)	(26)	(27)	(28)	(29)
18	Bachelors (ref. 12th grade or below)	1.00											
19	Masters	-0.36^*^	1.00										
20	Post-graduate degree	-0.34^*^	-0.17^*^	1.00									
21	Doctorate	-0.10^*^	-0.05^*^	-0.04	1.00								
22	Income (1- less than 20,000 Euros t- 7-150,000 Euros or more	-0.04	0.08^*^	0.15^*^	0.15^*^	1.00							
23	Middle management (ref. Administration; Board of Directors, Management and other Corporate Bodies)	0.06^*^	0.04	0.12^*^	-0.01	0.06^*^	1.00						
24	First line management	-0.01	-0.02	-0.06^*^	-0.05^*^	-0.17^*^	-0.31^*^	1.00					
25	No management position	-0.03	-0.07^*^	-0.10^*^	-0.03	-0.27^*^	-0.40^*^	-0.28^*^	1.00				
26	Tenure in the firm	-0.06^*^	-0.16^*^	-0.07^*^	0.07^*^	0.17^*^	-0.05^*^	-0.08^*^	-0.08^*^	1.00			
27	Log of sales	0.05^*^	-0.04	-0.04	-0.08^*^	-0.08^*^	0.07^*^	0.01	-0.02	-0.03	1.00		
28	Log of employees	0.03	0.01	0.06^*^	-0.08^*^	0.03	0.09^*^	0.02	-0.01	-0.07^*^	0.13^*^	1.00	
29	Net income (in ‘00,000 Euros)	0.03	0.00	0.03	-0.01	0.08^*^	0.02	0.02	0.00	0.01	0.10^*^	0.18^*^	1.00
30	Log of staff expenses	0.01	0.04	0.03	-0.06^*^	0.02	0.04	0.02	-0.02	-0.04	0.09^*^	0.80^*^	0.16^*^

*N*=1,897;

*p<0.05

The OLS estimates are presented in Table 21. As can be seen in the table, for OCBI in model 4 Behavioral QQ was negatively associated with OCBI (β =  -0.10, *p* <  0.01), in model 8 Emotional QQ was negatively associated with OCBO (β =  -0.04, *p* <  0.01), and in model 12 Behavioral QQ was negatively associated with turnover intentions (β =  -0.7, *p* <  0.05), however, Emotional QQ was positively related to turnover intentions (β =  0.4, *p* <  0.01).

**Table 21:** Study 4- OLS estimates.

	DV = OCBI				DV = OCBO				DV = Turnover Intention			
	(1)	(2)	(3)	(4)	(5)	(6)	(7)	(8)	(9)	(10)	(11)	(12)
QQ Behavioral		-0.12^***^		-0.10^***^		-0.04^**^		-0.02		0.02		-0.07^**^
		(0.03)		(0.03)		(0.02)		(0.02)		(0.02)		(0.03)
QQ Emotional			-0.07^***^	-0.02			-0.05^***^	-0.04^***^			0.11^***^	0.14^***^
			(0.02)	(0.02)			(0.01)	(0.01)			(0.02)	(0.03)
Voice	0.37^***^	0.35^***^	0.37^***^	0.35^***^	0.25^***^	0.24^***^	0.24^***^	0.24^***^	0.04	0.04	0.05	0.04
	(0.03)	(0.03)	(0.03)	(0.03)	(0.02)	(0.02)	(0.02)	(0.02)	(0.03)	(0.03)	(0.03)	(0.04)
Affective Commitment	0.06^***^	0.03^*^	0.05^***^	0.03^*^	0.06^***^	0.05^***^	0.06^***^	0.05^***^	-0.44^***^	-0.43^***^	-0.42^***^	-0.43^***^
	(0.02)	(0.02)	(0.02)	(0.02)	(0.02)	(0.02)	(0.02)	(0.02)	(0.03)	(0.03)	(0.03)	(0.03)
Continuance Commitment	0.00	0.01	0.02	0.02	0.01	0.02^**^	0.02^**^	0.02^**^	0.09^***^	0.09^***^	0.07^**^	0.07^**^
	(0.02)	(0.02)	(0.02)	(0.02)	(0.01)	(0.01)	(0.01)	(0.01)	(0.02)	(0.02)	(0.03)	(0.03)
Normative Commitment	0.08^***^	0.07^***^	0.08^***^	0.07^***^	-0.02	-0.02	-0.02	-0.02	-0.12^***^	-0.12^***^	-0.12^***^	-0.12^***^
	(0.02)	(0.02)	(0.02)	(0.02)	(0.02)	(0.02)	(0.02)	(0.02)	(0.02)	(0.02)	(0.02)	(0.02)
Perceived contract breach	-0.02	-0.02	-0.02	-0.02	0.01	0.01	0.01	0.01	0.22^***^	0.22^***^	0.22^***^	0.22^***^
	(0.02)	(0.02)	(0.02)	(0.02)	(0.01)	(0.01)	(0.01)	(0.01)	(0.02)	(0.02)	(0.02)	(0.02)
Feelings of violation	0.02	0.04^**^	0.03	0.04^**^	-0.05^**^	-0.04^*^	-0.04^*^	-0.04	0.32^***^	0.32^***^	0.3^***^	0.3^***^
	(0.02)	(0.02)	(0.02)	(0.02)	(0.02)	(0.02)	(0.02)	(0.02)	(0.02)	(0.02)	(0.02)	(0.02)
Social desirability	0.23^***^	0.22^***^	0.21^***^	0.21^***^	0.21^***^	0.21^***^	0.2^***^	0.2^***^	-0.03	-0.02	0.00	0.00
	(0.03)	(0.03)	(0.03)	(0.03)	(0.02)	(0.02)	(0.02)	(0.02)	(0.04)	(0.04)	(0.04)	(0.04)
Sex	-0.11^***^	-0.08^***^	-0.1^***^	-0.08^***^	-0.06^**^	-0.05^*^	-0.05^**^	-0.05^*^	0.05	0.05	0.04	0.05
	(0.02)	(0.02)	(0.02)	(0.02)	(0.02)	(0.02)	(0.02)	(0.02)	(0.06)	(0.06)	(0.06)	(0.06)
Age	-0.01	-0.01	-0.02	-0.01	0.02	0.03	0.02	0.02	0.00	0.00	0.01	0.01
	(0.03)	(0.03)	(0.03)	(0.03)	(0.03)	(0.03)	(0.03)	(0.03)	(0.04)	(0.04)	(0.04)	(0.04)
Married or in a union (ref. single)	0.03	0.02	0.02	0.02	-0.01	-0.01	-0.01	-0.01	0.00	0.00	0.01	0.00
	(0.04)	(0.03)	(0.04)	(0.03)	(0.03)	(0.03)	(0.03)	(0.03)	(0.04)	(0.04)	(0.04)	(0.04)
Divorced	-0.03	-0.03	-0.03	-0.03	0.01	0.01	0.01	0.01	-0.06	-0.06	-0.06	-0.06
	(0.06)	(0.05)	(0.06)	(0.05)	(0.04)	(0.04)	(0.04)	(0.04)	(0.07)	(0.07)	(0.07)	(0.07)
Widowed	-0.10	-0.09	-0.10	-0.10	-0.07	-0.07	-0.08	-0.07	-0.17	-0.17	-0.17	-0.16
	(0.16)	(0.14)	(0.15)	(0.14)	(0.19)	(0.18)	(0.18)	(0.18)	(0.24)	(0.24)	(0.25)	(0.25)
Bachelors (ref. 12th grade or below)	-0.06^**^	-0.06^*^	-0.06^*^	-0.05^*^	0.00	0.00	0.00	0.00	0.00	0.00	0.00	0.00
	(0.03)	(0.03)	(0.03)	(0.03)	(0.02)	(0.02)	(0.02)	(0.02)	(0.05)	(0.05)	(0.05)	(0.05)
Masters	-0.02	-0.01	-0.01	-0.01	0.01	0.01	0.02	0.02	0.06	0.06	0.04	0.04
	(0.05)	(0.05)	(0.05)	(0.05)	(0.04)	(0.04)	(0.04)	(0.04)	(0.09)	(0.09)	(0.09)	(0.09)
Post-graduate degree	-0.04	-0.03	-0.03	-0.03	0.03	0.03	0.03	0.03	0.13^*^	0.13^*^	0.13^*^	0.13^*^
	(0.04)	(0.04)	(0.04)	(0.04)	(0.03)	(0.03)	(0.03)	(0.03)	(0.07)	(0.07)	(0.07)	(0.07)
Doctorate	-0.02	0.00	-0.03	-0.01	0.06	0.07	0.06	0.06	-0.16	-0.17	-0.16	-0.14
	(0.08)	(0.07)	(0.08)	(0.07)	(0.10)	(0.10)	(0.10)	(0.10)	(0.16)	(0.16)	(0.16)	(0.16)
Income (1- less than 20,000 Euros t- 7-150,000 Euros or more	-0.01	-0.01	-0.01	-0.01	-0.01	-0.01	-0.01	-0.01	-0.04^**^	-0.04^**^	-0.03^**^	-0.03^**^
	(0.01)	(0.01)	(0.01)	(0.01)	(0.01)	(0.01)	(0.01)	(0.01)	(0.01)	(0.01)	(0.01)	(0.01)
Middle management (ref. Administration; Board of Directors, Management and other Corporate Bodies)	0.00	0.00	0.00	0.00	0.03	0.03	0.04	0.04	0.08^**^	0.08^**^	0.08^**^	0.07^*^
	(0.03)	(0.03)	(0.03)	(0.03)	(0.03)	(0.03)	(0.03)	(0.03)	(0.04)	(0.04)	(0.04)	(0.04)
First line management	0.06	0.07	0.06	0.07	0.06	0.07^*^	0.07^*^	0.07^*^	0.08	0.07	0.06	0.07
	(0.06)	(0.06)	(0.06)	(0.06)	(0.04)	(0.04)	(0.04)	(0.04)	(0.07)	(0.07)	(0.06)	(0.06)
No management position	0.06	0.08^*^	0.07	0.08^*^	0.09^**^	0.1^**^	0.1^**^	0.1^**^	-0.01	-0.02	-0.03	-0.02
	(0.05)	(0.04)	(0.04)	(0.04)	(0.04)	(0.04)	(0.04)	(0.04)	(0.05)	(0.05)	(0.05)	(0.05)
Tenure in the firm	0.00	0.00	0.00	0.00	0.00	0.00	0.00	0.00	0.00	0.00	0.00	0.00
	(0.00)	(0.00)	(0.00)	(0.00)	(0.00)	(0.00)	(0.00)	(0.00)	(0.00)	(0.00)	(0.00)	(0.00)
Log of sales	0.00	0.00	0.00	0.00	0.00	0.00	0.00	0.00	0.00	0.00	0.00	0.00
	(0.00)	(0.00)	(0.00)	(0.00)	(0.00)	(0.00)	(0.00)	(0.00)	(0.00)	(0.00)	(0.00)	(0.00)
Log of employees	-0.02	-0.02	-0.02	-0.02	0.01	0.02	0.02	0.02	0.05^**^	0.05^**^	0.05^*^	0.05^*^
	(0.02)	(0.01)	(0.02)	(0.01)	(0.01)	(0.01)	(0.01)	(0.01)	(0.03)	(0.03)	(0.03)	(0.03)
Net income (in ‘00,000 Euros)	0^**^	0.00	0^*^	0.00	0^***^	0^**^	0^***^	0^**^	0.00	0.00	0.00	0.00
	(0.00)	(0.00)	(0.00)	(0.00)	(0.00)	(0.00)	(0.00)	(0.00)	(0.00)	(0.00)	(0.00)	(0.00)
Log of staff expenses	0.01	0.01	0.01	0.01	-0.01	-0.01	-0.01	-0.01	-0.03^**^	-0.03^**^	-0.02^*^	-0.02^*^
	(0.01)	(0.01)	(0.01)	(0.01)	(0.01)	(0.01)	(0.00)	(0.00)	(0.01)	(0.01)	(0.01)	(0.01)
Constant	1.37^***^	1.73^***^	1.61^***^	1.76^***^	2.32^***^	2.45^***^	2.47^***^	2.51^***^	3.29^***^	3.23^***^	2.94^***^	3.03^***^
	(0.21)	(0.25)	(0.23)	(0.25)	(0.12)	(0.15)	(0.14)	(0.16)	(0.22)	(0.24)	(0.24)	(0.25)
Observations	1,897	1,897	1,897	1,897	1,897	1,897	1,897	1,897	1,897	1,897	1,897	1,897
R-squared	0.38	0.39	0.39	0.39	0.30	0.30	0.30	0.31	0.59	0.59	0.59	0.59
Adjusted R2	0.37	0.38	0.38	0.38	0.29	0.29	0.30	0.30	0.58	0.58	0.59	0.59

Standard errors clustered by 2-digit industry code.

***p < 0.01,

**p < 0.05,

*  *p* < 0.1.

As can be seen in Table 22, based on the results from dominance analysis, Behavioral QQ ranked 3^rd,^ and Emotional QQ ranked 6^th^ among the nine predictors explaining variation in OCBI. In terms of variance accounted for in the prediction of OCBO, both Behavioral and Emotional QQ ranked relatively in the mid-range, with Behavioral and Emotional QQ ranking 4^th^ and 5^th^, respectively. With respect to turnover intentions, the ranking for Behavioral QQ was 6^th^ of nine, and Emotional QQ emotional ranked 5^th^ of nine, above the relative impact of Social desirability, Voice, and continuance commitment. Based on the results from these meaningfulness rankings, relative to a number of strongly correlated constructs falling within the MQQS nomological network, we infer that Behavioral QQ is associated with lower levels of OCBI, while Emotional QQ is associated with lower levels of OCBO, and heightened levels of turnover intentions.

**Table 22:** Study 4 - dominance analysis.

	OCBI			OCBO			Turnover intention		
	Dominance Statistics	Std. Dominance Statistics (relative variance explained)	Ranking	Dominance Statistics	Std. Dominance Statistics (relative variance explained)	Ranking	Dominance Statistics	Std. Dominance Statistics (relative variance explained)	Ranking
Voice	0.16	0.42	1	0.12	0.41	1	0.01	0.02	7
Affective Commitment	0.03	0.09	4	0.02	0.08	3	0.16	0.27	1
Continuance Commitment	0.00	0.01	9	0.00	0.01	9	0.00	0.01	9
Normative Commitment	0.02	0.06	5	0.01	0.02	7	0.08	0.14	4
Perceived contract breach	0.01	0.03	7	0.01	0.02	8	0.12	0.20	3
Feelings of violation	0.01	0.02	8	0.01	0.04	6	0.12	0.21	2
Social desirability	0.08	0.20	2	0.09	0.29	2	0.01	0.01	8
QQ Behavioral	0.05	0.12	3	0.02	0.08	4	0.03	0.05	6
QQ Emotional	0.02	0.05	6	0.02	0.06	5	0.05	0.08	5
N	1,897			1,897			1,897		
Overall Fit Statistic	0.38			0.29			0.59		

### Summary

In our initial field test, we relied on the results from a Q-sort to identify a reduced set of items capturing the Behavioral and Emotional dimensions of the MQQS. In Study 2, leveraging a sample of Prolific participants, we aimed to explore the preliminary validity of the MQQS. Though dropping item 2 from the Behavioral QQ dimension led to a marginal improvement in model fit, the item represented a significantly lower loading. In Study 3, using a global sample of participants solicited from across the world, the results from analysis bearing on the structure of the MQQS did not support the removal of these scale items. However, in Study 4, the results from our analysis revealed that item seven of the Emotional QQ dimension could be dropped. Though the level of improvement in the scale fit was not significant, the item loadings improved. Across this series of four studies, the results from relative weight analysis indicated that the two dimensions of the MQQS showed high single digits, or low teens percent of relative variance explained in a set of conceptually endogenous, managerially relevant outcome variables.

Broadly, Behavioral QQ was associated with lower levels of OCBI, whereas Emotional QQ was associated with lower levels of OCBO and heightened turnover intentions. Based on the results from these four studies, the final MQQS with all items dropped is presented in Table A1. Hypothesis 1 was supported across all three field tests of the MQQS. The directions of effects predicted in Hypothesis 2 also were consistent with the direction of prediction for both OCBI and OCBO, and also revealed marginal support for turnover intentions in the regression analysis, and the relative weights analysis as well.

## Discussion

The goals of this study were to (a) provide an overview of prior conceptualizations and measures of various forms of workplace behavior reflective of diminished or limited contribution, (b) provide a multidimensional conceptualization of Quiet Quitting which was conceptually distinguished and does not overlap with other related constructs within its nomological network, and (c) develop and validate a multidimensional measure of Quiet Quitting. Following a review of adjacent construct definitions from research on diminished employee connection to work, and analysis of conceptualizations appearing in the literature we advanced a two-factor conceptualization of Quiet Quitting that included a behavioral and an emotional component. Building from this two-factor architecture, we developed and then validated a 13-item multidimensional quiet quitting scale, the MQQS, following a world-class scale development protocol. This multi-stage scale development process included collection of data from four separates samples, and the results from analysis based on these samples provided evidence of content, convergent, discriminant, criterion and incremental validity for the MQQS.

The essential elements of Quiet Quitting that emerged from our review of both focal and adjacent forms of diminished exchange contribution included a pattern of behavior reflective of an extreme minimum level of contribution [25,27], and an intense emotional reaction associated with deviations from this extreme lower-boundary pattern of exchange contribution [25,119]. The results from the scale development process described earlier provide evidence in support of the two-factor MQQS structure we describe, across three separate field samples. The MQQS also repeatedly demonstrated consistently high levels of fit, and a high level of psychometric validity. Development of a valid, multidimensional measure of Quiet Quitting that coincides with current depictions in the literature opens the door for academic researchers and practitioners alike to examine relationships between the construct and a range of workplace factors with the potential diminish the incidence of this dangerous pattern of employee behavior. For example, our analysis revealed that while Behavioral QQ Behavioral is negatively related to OCBI, Emotional QQ is negatively related to OCBO. Though QQ Behavioral is negatively related to turnover intentions, Emotional QQ is more positively related to turnover intention. The results reported across all four studies (detailed results are reported on OSF) provide strong support for the dimensionality of the MQQS, and thus represent an opportunity to develop dimension specific understanding of the operation of the construct. This paves the way for more conceptually anchored theoretical development in the domain.

Our findings extend organizational behavior theory in several important ways. First, while social exchange theory has traditionally focused on reciprocity in workplace relationships [71], our two-dimensional conceptualization of Quiet Quitting suggests that withdrawal behaviors may have both behavioral and emotional reciprocity components. This aligns with research on psychological contract violations [64] but extends it by showing how employees actively calibrate both their behavioral contributions and emotional investments.

The strong relationship we found between behavioral Quiet Quitting and reduced OCBIs parallels findings on withdrawal behaviors [53] but suggests a more nuanced process where employees strategically withdraw discretionary behaviors while maintaining minimal task performance. Similarly, emotional Quiet Quitting’s relationship with turnover intentions extends understanding of how psychological withdrawal [120] may precede physical withdrawal, though through a previously unexamined pathway of intentional emotional detachment.

Our findings across multiple samples suggest Quiet Quitting represents a distinct form of workplace withdrawal that differs from established constructs like burnout [35] or psychological contract breach [37]. While these constructs often reflect reactions to workplace conditions, quiet quitting appears to represent a proactive strategy for managing work investment, suggesting important theoretical implications for models of employee engagement and withdrawal.

The validation of MQQS across different cultural contexts (Studies 3 and 4) extends recent work on cross-cultural differences in work values [9] and suggests Quiet Quitting may manifest differently across cultural boundaries. This has important implications for international management theory and practice, particularly as organizations manage increasingly global workforces with different cultural norms regarding work contribution.

## Limitations and future research directions

This research is not without limitations. Quiet Quitting is an emerging sociological phenomenon. One limitation of exploring this phenomenon is understanding how well entrenched this behavior may become over time. As depicted in the popular press, even large firms are facing significant challenges in motivating employees in the post-pandemic world. Rising levels of automation and wage pressures most likely may lead to an increased prevalence of Quiet Quitting. Though our study relies on self-reports, future research could focus on the impact of Quiet Quitting on the productivity of firms. There are growing concerns related to negative career and organizational implications of Quiet Quitting. However, Quiet Quitting also may lead to improved employee health and work-life balance. These countervailing forces may be an important consideration in understanding the implications of Quiet Quitting, and momentum in support of not prematurely framing MQQS as either an exclusively positive or negative organizational phenomenon.

Second, though we used a gamut of theoretically validated constructs to assess the nomological and criterion validity of the MQQS, we do not claim to have fully addressed the omitted variable bias. However, the relative variance explained by our proposed scale is meaningful. Future research could meaningfully focus on the implications of related constructs as understanding of this construct continues to emerge. A sister and a complementary construct is Quiet Hiring. How employers manage challenges associated with Quiet Quitting is an important consideration in understanding how employers are actively responding to this phenomenon, and the potential spillover implications for important organizational outcomes.

Third, though our samples were generated from the United States and also from countries across the world, generalizability concerns cannot be ruled out. In other countries, there may be work behaviors that parallel patterns reflected by Quiet Quitting. These cross-cultural variations represent an important consideration for future researchers to adopt. For example, in high power distance cultures, or those characterized by high levels of collectivism, Quiet Quitting may be less plausible; whereas in cultures characterized by higher levels of individualism the prevalence and implications of the phenomenon may be stronger and more prevalent.

Fourth, though much of the focus orbiting the Quiet Quitting phenomenon has been framed at the individual-level, a rich set of studies on network-related spillovers represent a potentially important point of future focus. Though Quiet Quitting may seemingly be an individual choice, its contagion, or lack thereof in a given workplace has rich theoretical and practical implications. For example, the question of how teams or work units cope with Quiet Quitters emerges as a non-trivial consideration within organizations increasingly defined by the presence of collectives such as groups and teams. Another important question orbits the issue of whether the context (e.g., high-pressure workplaces or tasks with higher levels of discretion) impact the emergence or outcomes associated with Quiet Quitting. What also emerges are questions relating to the prevalence of Quiet Quitting across different sectors, and whether certain employee-types attracted to certain occupations and industries may be more prone to Quiet Quitting. It will also be important to explore how the network position of a Quiet Quitter affects the outcomes of others embedded within the social network. These represent the tip of the iceberg of important micro-, meso-, and firm-level considerations for future research.

Finally, while our studies demonstrate the MQQS’s validity across multiple samples, including international respondents, future research should explicitly test for measurement invariance across cultural contexts. This would involve collecting adequate sample sizes from distinct cultural contexts, translating and back-translating the MQQS into multiple languages, and conducting formal tests of configural, metric, and scalar invariance across cultures. Such analyses would enhance our understanding of how Quiet Quitting manifests across cultural boundaries and whether the construct operates equivalently in different cultural contexts. This is particularly important given that work norms, emotional display rules, and acceptable levels of minimum contribution may vary significantly across cultures. For example, in high power distance cultures, or those characterized by high levels of collectivism, Quiet Quitting may be less plausible; whereas in cultures characterized by higher levels of individualism the prevalence and implications of the phenomenon may be stronger and more prevalent. Future research should examine whether the behavioral and emotional dimensions of Quiet Quitting manifest differently across cultural contexts, particularly in cultures with different norms regarding work contribution and emotional expression. These cross-cultural variations represent an important consideration for future researchers to adopt, especially as organizations become increasingly global and diverse in their workforce composition.

Our findings suggest several promising directions for future research that directly address current limitations. First, while our cross-sectional design provides initial validation of the MQQS, longitudinal research is crucial for understanding how Quiet Quitting develops and evolves over time. Such temporal studies could employ experience sampling methodologies to capture daily fluctuations in Quiet Quitting behaviors and emotions, while multi-source data including supervisor ratings and objective performance metrics would overcome limitations of self-report measures.

Second, our findings about Quiet Quitting’s potential effects on employee wellbeing warrant deeper investigation. Researchers should examine both positive and negative outcomes using objective measures of physiological and psychological health. This could reveal whether Quiet Quitting serves as an adaptive strategy for maintaining work-life balance or leads to deteriorating professional relationships and career outcomes.

Third, while our global sample provides initial evidence of the MQQS’s cross-cultural applicability, future research should explicitly examine cultural and contextual boundary conditions. This includes formal measurement invariance testing across cultures, investigation of how national culture dimensions moderate Quiet Quitting manifestations, and analysis of industry-specific contexts that might influence Quiet Quitting’s prevalence and impact. Fourth, the network effects of Quiet Quitting deserve attention. Research using social network analysis could reveal how Quiet quitting behaviors spread within work units, while matched employer-employee data could illuminate organizational performance implications. Understanding these contagion effects is particularly important given increasing workforce virtualization and changing workplace dynamics. Finally, intervention-focused research is needed to help organizations effectively manage Quiet Quitting. This includes studying how leadership styles and organizational culture influence Quiet Quitting, developing and testing intervention strategies, and examining Quiet Quitting’s relationship with newer forms of work arrangements such as remote work and the gig economy. Such research would help translate our theoretical understanding into practical organizational solutions.

## Conclusion

Quiet Quitting encompasses a pattern of employee work behavior reflective of an extreme minimum level of exchange contribution with their organization at a level that only just allows the employee to avoid involuntary turnover; coupled with an extreme negative reaction to contributions that extend beyond this asymptotic lower-level exchange boundary. In this research, we develop and validate a novel measure of Quiet Quitting – the MQQS – which captures these aspects of the Quiet Quitting construct. Quiet Quitting is increasingly prevalent among Millennials and Generation Z employees and represents a potentially extremely dangerous pattern of employee work-place contribution in light of growing evidence for the importance of employee contributions extending beyond minimum levels of workplace compliance [25,52,53], and the fragility of organizations where employees provide only contractually defined contributions [54,55]. The results from this research provide a lever for managers and researchers, advancing understanding of how the dimensions of Quiet Quitting relate to important workplace outcomes. The results from the analysis we report provide evidence that MQQS is distinct from – and provides significant marginal incremental predictive insight into – key outcomes and conceptually adjacent measures of employee contribution. The results we report also demonstrate the importance of a multi-dimensional conceptualization of Quiet Quitting which reveals nuanced associations with outcomes such as OCBI, OCBO, and turnover intentions. This new measure should provide scholars and managers alike with an important means to advance understanding of Quiet Quitting in modern organizations.

## Supporting information

S1 Appendix Table A1. Quiet quitting scale. (DOCX)
