# Supplementary material for: A multidimensional quiet quitting scale: Development and test of a measure of quiet quitting
Source: PLoS One. 2025 Apr 15;20(4):e0317624. doi: 10.1371/journal.pone.0317624 (PMC11999150; doi:10.1371/journal.pone.0317624)
Supplement: S1 Appendix — (DOCX) [file pone.0317624.s001.docx]

**Appendix**

**Table A1. *Quiet Quitting scale***

| **Quiet Quitting (14 item, 1-6 behavioral, 7-13 emotional)**  1= strongly disagree 5 = strongly agree | | | | | | |  |  |  |  |
| --- | --- | --- | --- | --- | --- | --- | --- | --- | --- | --- |
| **Behavioral Quiet Quitting** |  |  |  |  |  | | |  | |  |
| 1. I do only the work I’m specifically asked to do; just enough to not lose my job. | 1 | 2 | 3 | 4 | | 5 | | |  | |
| 2. I spend the adequate time necessary working on tasks to keep my job.* | 1 | 2 | 3 | 4 | | 5 | | |  | |
| 3. I do not do extra work beyond what I’m paid to do. | 1 | 2 | 3 | 4 | | 5 | | |  | |
| 4. I don’t look for extra work to do even though it could help me to get promoted. | 1 | 2 | 3 | 4 | | 5 | | |  | |
| 5. To keep my job, I believe in working just enough, not harder or smarter. | 1 | 2 | 3 | 4 | | 5 | | |  | |
| 6. Doing only the work that is required is smart, not lazy. | 1 | 2 | 3 | 4 | | 5 | | |  | |
| **Emotional Quiet Quitting** |  |  |  |  |  | | |  | |  |
| 7. It doesn’t bother me when extra work gets left unfinished. | 1 | 2 | 3 | 4 | 5 | | |  | |  |
| 8. It is emotionally rewarding for me not looking for extra work to do. | 1 | 2 | 3 | 4 | 5 | | |  | |  |
| 9. Worries weigh heavily on my mind, when going above and beyond in my work. | 1 | 2 | 3 | 4 | 5 | | |  | |  |
| 10. My emotions often multiply negatively when working beyond what is necessary to keep my job. | 1 | 2 | 3 | 4 | 5 | | |  | |  |
| 11. I feel less burned out by doing only the work that is needed to keep my job. | 1 | 2 | 3 | 4 | 5 | | |  | |  |
| 12. My mental health is a lot better by not going the ‘extra mile’ at work. | 1 | 2 | 3 | 4 | 5 | | |  | |  |
| 13. I don’t tend to spend time mulling over work-related issues.^ | 1 | 2 | 3 | 4 | 5 | | |  | |  |

*Dropped in Study 1

^ Dropped in Study 3
